# Voltage-dependent calcium channel signaling mediates GABA_A_ receptor-induced migratory activation of dendritic cells infected by *Toxoplasma gondii*

**DOI:** 10.1371/journal.ppat.1006739

**Published:** 2017-12-07

**Authors:** Sachie Kanatani, Jonas M. Fuks, Einar B. Olafsson, Linda Westermark, Benedict Chambers, Manuel Varas-Godoy, Per Uhlén, Antonio Barragan

**Affiliations:** 1 Department of Molecular Biosciences, The Wenner-Gren Institute, Stockholm University, Stockholm, Sweden; 2 Center for Infectious Medicine, Department of Medicine, Karolinska University Hospital Huddinge, Karolinska Institutet, Stockholm, Sweden; 3 Laboratory of Molecular Neurobiology, Department of Medical Biochemistry and Biophysics, Karolinska Institutet, Solna, Sweden; 4 Centro de Investigacion Biomedica, Faculty of Medicine, Universidad de los Andes, Santiago, Chile; University of New Mexico, UNITED STATES

## Abstract

The obligate intracellular parasite *Toxoplasma gondii* exploits cells of the immune system to disseminate. Upon *T*. *gondii*-infection, γ–aminobutyric acid (GABA)/GABA_A_ receptor signaling triggers a hypermigratory phenotype in dendritic cells (DCs) by unknown signal transduction pathways. Here, we demonstrate that calcium (Ca^2+^) signaling in DCs is indispensable for *T*. *gondii-*induced DC hypermotility and transmigration *in vitro*. We report that activation of GABA_A_ receptors by GABA induces transient Ca^2+^ entry in DCs. Murine bone marrow-derived DCs preferentially expressed the L-type voltage-dependent Ca^2+^ channel (VDCC) subtype Ca_v_1.3. Silencing of Ca_v_1.3 by short hairpin RNA or selective pharmacological antagonism of VDCCs abolished the Toxoplasma-induced hypermigratory phenotype. In a mouse model of toxoplasmosis, VDCC inhibition of adoptively transferred *Toxoplasma*-infected DCs delayed the appearance of cell-associated parasites in the blood circulation and reduced parasite dissemination to target organs. The present data establish that *T*. *gondii*-induced hypermigration of DCs requires signaling via VDCCs and that Ca^2+^ acts as a second messenger to GABAergic signaling via the VDCC Ca_v_1.3. The findings define a novel motility-related signaling axis in DCs and unveil that interneurons and DCs share common GABAergic motogenic pathways. *T*. *gondii* employs GABAergic non-canonical pathways to induce host cell migration and facilitate dissemination.

## Author summary

Dendritic cells are considered the gatekeepers of the immune system but can, paradoxically, also function as ‘Trojan horses’ to mediate dissemination of the common intracellular parasite *Toxoplasma gondii*. Previous work has shown that *Toxoplasma* hijacks the migratory machinery of dendritic cells by inducing secretion of the neurotransmitter GABA and by activating GABAergic signaling pathways, thereby making infected dendritic cells hypermigratory *in vitro* and *in vivo*. Here, we show that the signaling molecule calcium plays a central role for this migratory activation and that signal transduction is preferentially mediated through a subtype of voltage-gated calcium channel (Ca_v_1.3). This study functionally implicates Ca_v_1.3 channels in a, hitherto uncharacterized, calcium signaling axis by which dendritic cells are induced to become migratory. The studies show how an obligate intracellular pathogen takes advantage of non-canonical signaling pathways in immune cells to modulate their migratory properties, and thereby facilitate the dissemination of the parasite.

## Introduction

The obligate intracellular parasite *Toxoplasma gondii* chronically infects a large portion of the global human population and is capable of infecting any warm-blooded vertebrate [1]. The dissemination of the parasite from the point of entry in the intestinal tract plays a determinant role in the pathogenesis of toxoplasmosis. Although chronic infection is generally considered asymptomatic in otherwise healthy individuals, reactivated infection in the central nervous system (CNS) of immune-compromised individuals may be fatal. Congenital toxoplasmosis occurs by transmission to the fetus from the infected mother and can result in serious disabilities or death of the unborn child [2].

Previous studies have demonstrated that active invasion of dendritic cells (DCs) by *T*. *gondii* tachyzoites rapidly (within minutes) induces a hypermigratory phenotype in parasitized DCs [3]. This migratory activation is characterized by cytoskeletal rearrangements and dramatically enhanced cellular locomotion, termed hypermotility [4], and enhanced transmigratory activity *in vitro* [5]. These phenotypes have been linked to enhanced dissemination and parasitic loads in mice for different species of apicomplexan parasites [5–7]. The initiation of the hypermigratory phenotype in DCs is related to the discharge of secretory organelles during parasite invasion and does not depend on *de novo* protein synthesis in the host cell [4]. It is mediated through non-canonical GABAergic signaling pathways, and is independent of MyD88-mediated TLR signaling and chemotaxis [3–5, 8].

Within the context of the host-parasite interaction, we have recently shown that DCs possess functional GABA_A_ receptors, and the capability to synthesize and secrete γ–aminobutyric acid (GABA) [8]. Challenge with *T*. *gondii* triggered GABA secretion in the invaded DCs and inhibition of GABA_A_ receptors, GABA synthesis or GABA transport abrogated the *T*. *gondii*-induced hypermigratory phenotype [8]. Along these lines, mounting evidence shows that GABA, the main inhibitory neurotransmitter in the vertebrate brain, participates outside the CNS in diverse functions including cell migration, immunomodulation and metastasis [9–11].

GABA_A_ receptors are ionotropic chloride channels whose functions are regulated by cation-chloride co-transporters [12]. Membrane depolarization secondary to GABA receptor activation can elicit opening of voltage-dependent Ca^2+^ channels (VDCCs, also termed voltage-gated Ca^2+^ channels, VGCCs) that are normally closed at physiologic or resting membrane potential [13–15]. Thus, GABA-mediated Ca^2+^ influx via VDCCs is a well-established concept in neuronal cells but remains unexplored in immune cells. While various Ca^2+^ signaling pathways have been implicated in the regulation of multiple DC functions, including activation, maturation and formation of immunological synapses with T cells (reviewed in [16]), knowledge on the role of VDCCs in DCs remains limited [17, 18].

Here, we show how an obligate intracellular pathogen takes advantage of a hitherto uncharacterized Ca^2+^ signaling axis in DCs to modulate the migration of parasitized host cells. We demonstrate that the hypermigratory phenotype induced in DCs by *T*. *gondii* is predominantly dependent on the L-type VDCC subtype Ca_v_1.3, which is activated by GABAergic signaling upon *T*. *gondii* invasion.

## Results

### *T*. *gondii-*induced DC hypermotility and transmigration depend on physiological extracellular Ca^2+^ concentrations

**Fig 1:**
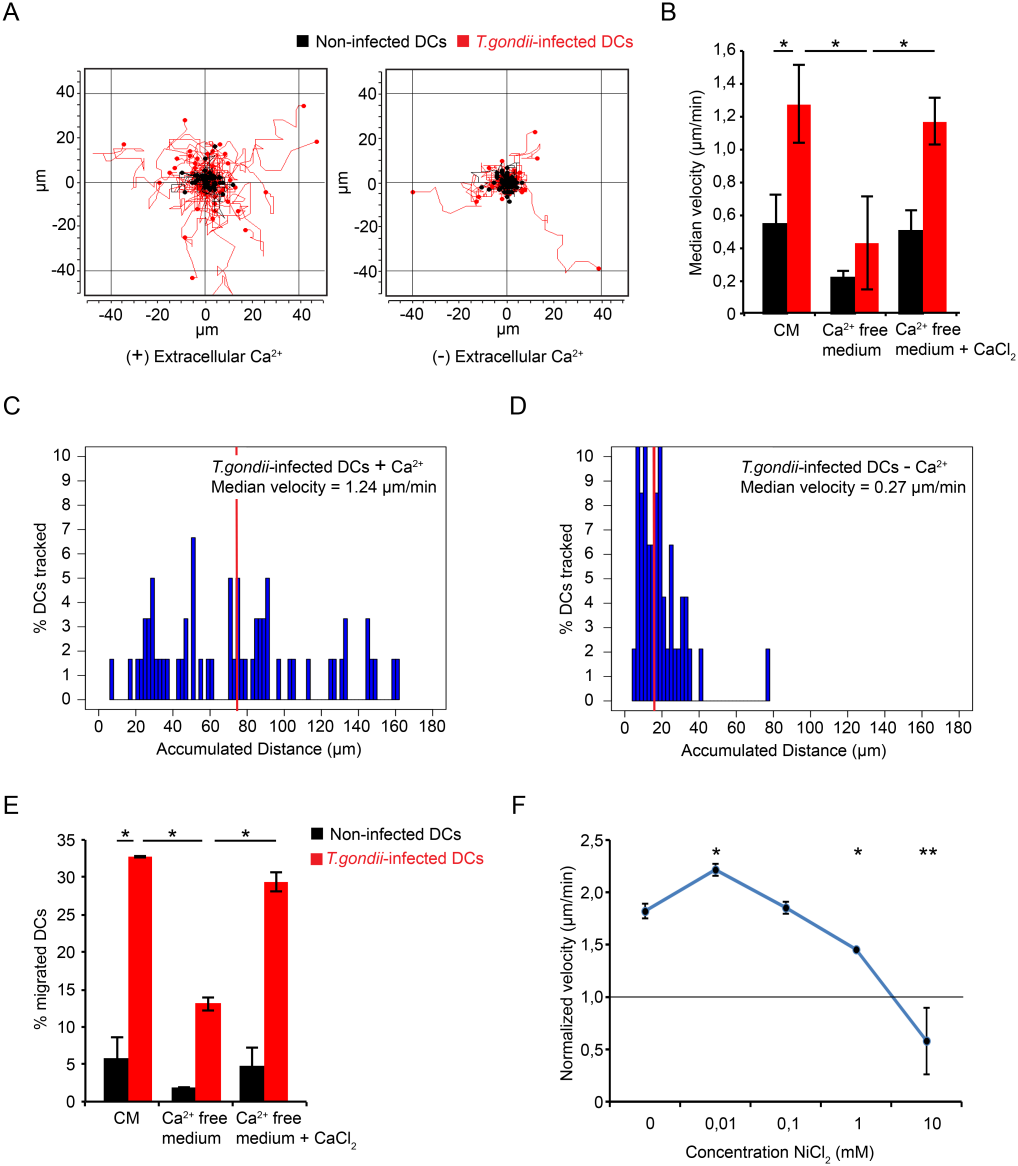
DC hypermotility and transmigration are abrogated at low extracellular Ca^2+^ concentration. **(A)** Representative motility plot analysis of DCs challenged with *T*. *gondii* in Ca^2+^-free medium, 1% FBS ± CaCl_2_ (1.8 mM). DCs were pre-incubated with freshly egressed *T*. *gondii* PRU-RFP tachyzoites (MOI 3, 4 h) in complete medium (CM) as described in Materials and Methods. Red and black track plots indicate infected DCs (RFP^+^) and non-infected DCs (RFP_-_), respectively. **(B)** Bar graph represents median velocity of DCs from 3 independent experiments (*n* = 60 cells per experiment) performed as in (A). Asterisks indicate significant differences (*: p < 0.001, Pairwise Wilcoxon rank-sum test, Holm correction). **(C and D)** Histograms show distributions of accumulated distances migrated by *T*. *gondii*-infected DCs in the presence (C) or absence (D) of extracellular Ca^2+^. Vertical red lines indicate, for each condition, the median distance migrated by cells. Significant differences in distances migrated were observed between the conditions (p < 0.001, Wilcoxon rank-sum test, Holm correction). Data are representative of 3 independent experiments. **(E)** Transmigration frequency of DCs incubated with freshly egressed *T*. *gondii* tachyzoites (MOI 3) for 6 h in CM. Transmigration assay was performed in Ca^2+^-free medium or in CM as described under Materials and Methods. Data represent means (± SD) of 3 independent experiments. Asterisks indicate significant differences (*: p < 0.01, One-way ANOVA, Tukey’s HSD test). **(F)** Normalized velocity of DCs incubated with freshly egressed *T*. *gondii* PRU tachyzoites (MOI 3) for 3 h in CM and treated with NiCl_2_ for 1 h. Data represent median velocities (± SD) from 2 independent experiments (*n* = 60 cells per experiment) normalized against a non-infected control (horizontal line). Asterisks indicate significant differences vs. non-treated control (*: p < 0.05, **: p < 0.001, Steel’s Many-one Rank test, Holm correction).

Shortly after parasite entry, DCs exhibit a dramatic migratory activation [5, 8]. Based on the implication of GABAergic signaling and on the rapid onset of the hypermigratory phenotype minutes after parasite invasion [4], we hypothesized a role for Ca^2+^ signal transduction for the induction and maintenance of *T*. *gondii-*induced migration. When DC motility was assessed in a Ca^2+^-deprived medium, individual cell track analysis of infected DCs showed a reduction in migrated distances (Fig 1A) and a significant downward shift in the distribution of migrated distances (Fig 1C and 1D). In low Ca^2+^ medium with 1% FBS, the median velocity of *T*. *gondii-*infected DCs was significantly reduced, and addition of Ca^2+^ (CaCl_2_) at physiological concentration reconstituted hypermotility in infected DCs (Fig 1B). Similarly, base-line motility of non-challenged DCs was reduced upon Ca^2+^ deprivation and reconstituted by addition of Ca^2+^ (Fig 1B and S1 Fig). In line with motility assays, the relative transmigration frequencies of infected DCs, and non-infected DCs, across a transwell porous-membrane were significantly reduced in low extracellular Ca^2+^ and reconstituted upon addition of Ca^2+^ at physiological concentration (Fig 1E). Next, we assessed the motility of infected DCs in the presence of NiCl_2_, which blocks plasma membrane Ca^2+^ channels. NiCl_2_ dose-dependently reduced the velocity of hypermotile infected DCs, that reached velocities comparable with non-infected DCs (Fig 1F). Altogether, the present data indicate that the *T*. *gondii*-induced hypermigratory phenotype of DCs is dependent on the entry of extracellular Ca^2+^ through plasma membrane Ca^2+^ channels.

### Stimulation of DCs with GABA elicits Ca^2+^ influx transients in the DC cytosol

**Fig 2:**
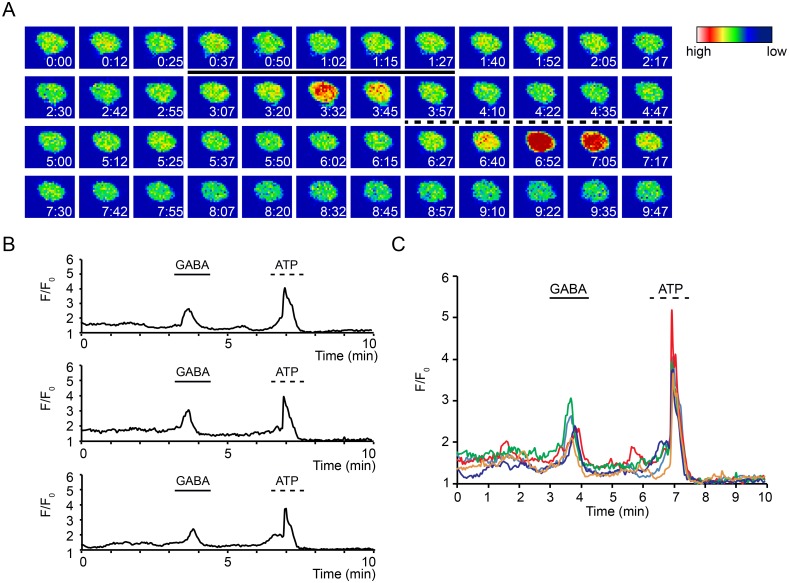
GABA elicits Ca^2+^ influx transients in DCs. **(A)** Representative pseudocolor micrographs of live cell Ca^2+^ imaging of DCs loaded with 2 μM Fluo-8H/AM as described in Materials and Methods. Continuous line indicates perfusion time of GABA (10 mM) and dashed line indicates perfusion time of ATP (50 μM). For each micrograph color scale indicate fluorescence intensity at indicated time point (min:sec). **(B)** Relative fluorescence intensity of DCs as in (A). The lines indicate perfusions of GABA (10 mM) after 3 min and ATP (50 μM) after 6.5 min, respectively. Displayed data are 3 representative traces from one experiment. **(C)** Relative fluorescence intensity of DCs as in (A) and (B). Displayed data are 5 superposed representative traces from the same experiment. Experiments were performed 3 times with similar results (n = 15–20 cells per experiment).

We have previously established that infection by *T*. *gondii* induces motility-related GABAergic signaling pathways in DCs [8]. Because hypermotile Toxoplasma-infected DCs exhibited dependency on Ca^2+^ and the established links between GABA receptor activation and Ca^2+^ responses in neuronal cellular systems [13, 14], we tested whether GABA_A_ receptor activation led to Ca^2+^ responses in DCs. Perfusion of GABA elicited cytosolic Ca^2+^ elevations in DCs, visualized by fluorescent Ca^2+^ indicators (Fig 2A and S1 Video). Stimulation of DCs with GABA led to a simultaneous and transient Ca^2+^ influx (Fig 2B and 2C) in ~ 20% of the tested DC population at a given time point and, for the reference stimulus ATP, in ~ 42% of DCs (S1 Table). Ca^2+^ transients induced by GABA had relatively similar longevity and relatively lower amplitude than responses to ATP (Fig 2B and 2C), which were in line with ATP responses previously characterized in various types of DCs [19, 20]. Upon repeated stimulations with GABA and at varying GABA concentrations, consecutive Ca^2+^ responses were observed in individual cells (S2 Fig). Altogether, the data is in line with the previously recorded GABA-induced membrane potential changes by patch-clamping [8] and demonstrate that GABA stimulation of DCs is followed by influx of Ca^2+^ and transiently increased cytosolic Ca^2+^ concentration.

### Ca^2+^ channel agonism reconstitutes hypermigration in GABA-inhibited Toxoplasma-infected DCs

**Fig 3:**
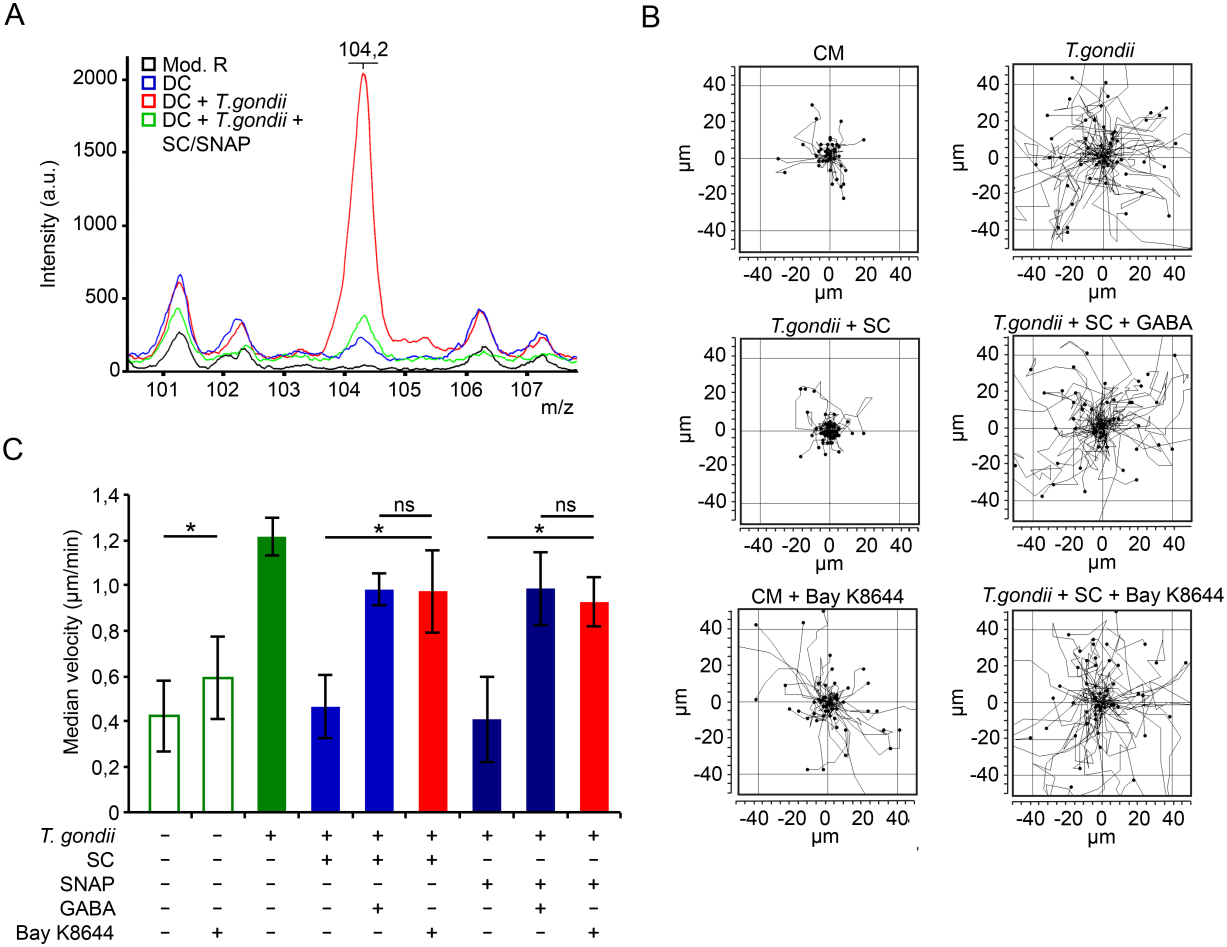
Exogenous GABA and Ca^2+^ receptor agonism reconstitutes DC hypermotility. **(A)** Mass spectrometry analysis of DCs challenged with tachyzoites (PRU, MOI 3) for 16 h ± GABAergic inhibition (SC, 50 μM; SNAP, 50 μM). Cell supernatant was analyzed as indicated under Materials and Methods. Mod. R indicates modified Krebs-Ringer’s solution. Data are representative of 3 independent experiments. **(B)** Representative motility plot analyses of DCs incubated with tachyzoites (PTG, MOI 3) for 2 h with SC (50 μM), SNAP (50 μM), ± GABA (5 μM) or Bay K8644 (10 μM). **(C)** Compiled motility analysis of DCs under same conditions as in (B). Motility assays were performed as described under Materials and Methods. Data represent median velocities ± SD of 3 independent experiments. Asterisks indicate significant differences (*: p < 0.01, ns: p ≥ 0.05, Pairwise Wilcoxon rank-sum test, Holm correction).

Next, we sought to determine if the GABA-induced Ca^2+^ signaling in Toxoplasma-infected DCs had an impact on hypermotility. First, we determined GABA secretion by infected DCs and the deprivation of GABA upon pre-incubation with GABAergic inhibitors (SC/SNAP) using MALDI mass spectrometry analysis of cell supernatants. In supernatants from Toxoplasma-infected DCs, spectra displayed a distinct peak signal (*m/z* 104,2; Fig 3A) corresponding to the signal of protonated GABA [M + H]^+^ chemical grade analytical standard (S3 Fig) [21]. Inhibition of GABA synthesis and secretion (SC, SNAP inhibitors, respectively) selectively reduced the *m/z* 104,2 peak signal (Fig 3A) and abrogated the hypermotility of *T*. *gondii*-infected DCs, which was reconstituted by addition of exogenous GABA (Fig 3B and 3C). This provided further specificity to previously reported elevations of GABA secretion in Toxoplasma-infected DCs, as quantified by GABA-ELISA under the same conditions [8]. Next, to test the impact of Ca^2+^ influx in DC hypermotility under GABA-deprived conditions, a cell membrane Ca^2+^ channel/ L-type VDCC agonist (BayK8644) was added to the cells. Importantly, the abrogated hypermotility of infected DCs, generated by GABAergic inhibition, was rescued by addition of BayK8644 (Fig 3B and 3C). A moderate but significant increase in cell motility was also observed in naïve DCs in presence of BayK8644 (Fig 3B and 3C). We conclude that, upon GABAergic inhibition, Ca^2+^ channel (VDCC) agonism leading to Ca^2+^ entry in DCs can reconstitute hypermotility in Toxoplasma-infected DCs.

### Upon inhibition of voltage-dependent Ca^2+^ channels (VDCCs), exogenous GABA fails to reconstitute hypermigration in Toxoplasma-infected DCs

**Fig 4:**
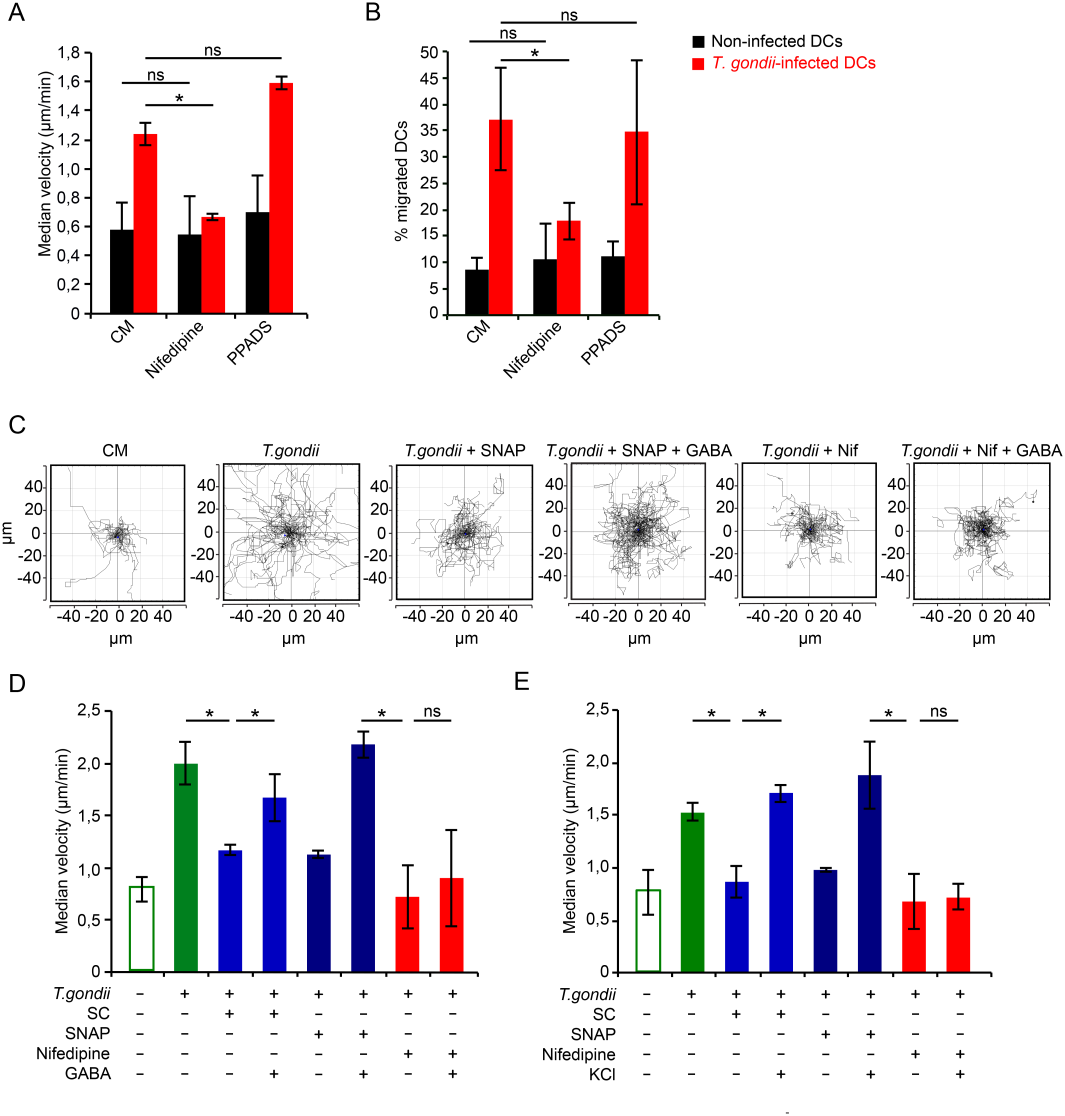
VDCC blockade abolishes GABA-mediated hypermigration of infected DCs that is not rescued by exogenous GABA or membrane depolarization. **(A)** Motility analysis of DCs incubated with PRU tachyzoites for 3 h and treated for 1 h with nifedipine (10 μM) or PPADS (100 μM). Data represent median velocities ± SD of 3 independent experiments. Asterisks indicate significant differences (*: p < 0.001, ns: p ≥ 0.05, Pairwise Wilcoxon rank-sum test, Holm correction). **(B)** Transmigration frequency of DCs challenged with PRU tachyzoites for 5 h followed by treatment (1 h) with nifedipine (30 μM) or PPADS (100 μM). Data represent means ± SD of 3 independent experiments performed in duplicate. Asterisks indicate significant differences (*: p < 0.01, ns: p ≥ 0.05, One-way ANOVA, Tukey’s HSD test). (**C)** Representative motility plot analyses of DCs incubated with tachyzoites (PRU, MOI 3) for 2h with SNAP (50 μM), ± GABA (5 μM) or nifedipine (Nif, 10μM) ± GABA (5 μM). **(D)** Motility analysis of DCs incubated with PRU tachyzoites and treated for 2 h with SC (50 μM), SNAP (50 μM) or nifedipine (10 μM), ± GABA (5 μM). Data represent median velocities ± SD of 3 independent experiments. Asterisks indicate significant differences (*: p < 0.001, ns: p ≥ 0.05, Pairwise Wilcoxon rank-sum test, Holm correction). **(E)** Motility analysis of DCs incubated with PRU tachyzoites and treated as in (D) ± KCl (25 mM). Data represent median velocities ± SD of 3 independent experiments. Asterisks indicate significant differences (*: p < 0.001, ns: p ≥ 0.05, Pairwise Wilcoxon rank-sum test, Holm correction).

VDCCs are known to respond with Ca^2+^ permeability to membrane potential changes. Because GABA_A_ receptor activation by GABA elicits membrane potential changes in Toxoplasma-infected DCs [8] and GABA elicited Ca^2+^ influx (Fig 2), we investigated the putative involvement of VDCCs in DC hypermotility. L-type VDCC inhibition by nifedipine abolished hypermotility (Fig 4A) and significantly reduced transmigration (Fig 4B). In sharp contrast, inhibition of purinergic Ca^2+^ receptors by PPADS at high concentrations [22] had non-significant effects on hypermotility and transmigration of infected DCs (Fig 4A and 4B), despite that activation of purinergic receptors by ATP caused a significant Ca^2+^ influx and increased cytosolic Ca^2+^ levels in DCs (Fig 2). This indicated that VDCC-related effects governed hypermigration. We therefore explored further the function of VDCCs in relation to GABAergic signaling.

We previously reported that inhibition of GABA synthesis (SC) and/or transport (SNAP) significantly reduced GABA secretion and transmigration of *T*. *gondii*-infected DCs [8]. Extending these observations, addition of exogenous GABA rescued the hypermotility of infected DCs under GABAergic inhibition (Fig 4C and 4D). In sharp contrast, VDCC inhibition by nifedipine treatment caused a significant decrease in the motility of infected DCs that was not restored by exogenous GABA (Fig 4C and 4D), indicating implication of L-type VDCCs downstream of GABAergic signaling.

At resting membrane potential VDCCs are normally closed and, respond with Ca^2+^ permeability upon membrane depolarization. To relate the effect of GABAergic signaling to that of membrane depolarization, we treated GABA-deprived infected DCs with the depolarizing agent KCl. Upon blockade of GABA synthesis and secretion, KCl treatment fully restored hypermotility in Toxoplasma-infected DCs (Fig 4E), thus mimicking the effects obtained by addition of exogenous GABA (Figs 4D and 3C). Importantly, hypermotility was not restored by KCl in the presence of the L-type VDCC inhibitor nifedipine (Fig 4E). Taken together with the effects of VDCC agonism (Fig 3), these data demonstrate a link between L-type VDCCs and the hypermigratory phenotype of *T*. *gondii*-infected DCs downstream of GABAergic signaling.

### The dominantly expressed VDCC, Ca_v_1.3, is linked to hypermigration of Toxoplasma-infected DCs

**Fig 5:**
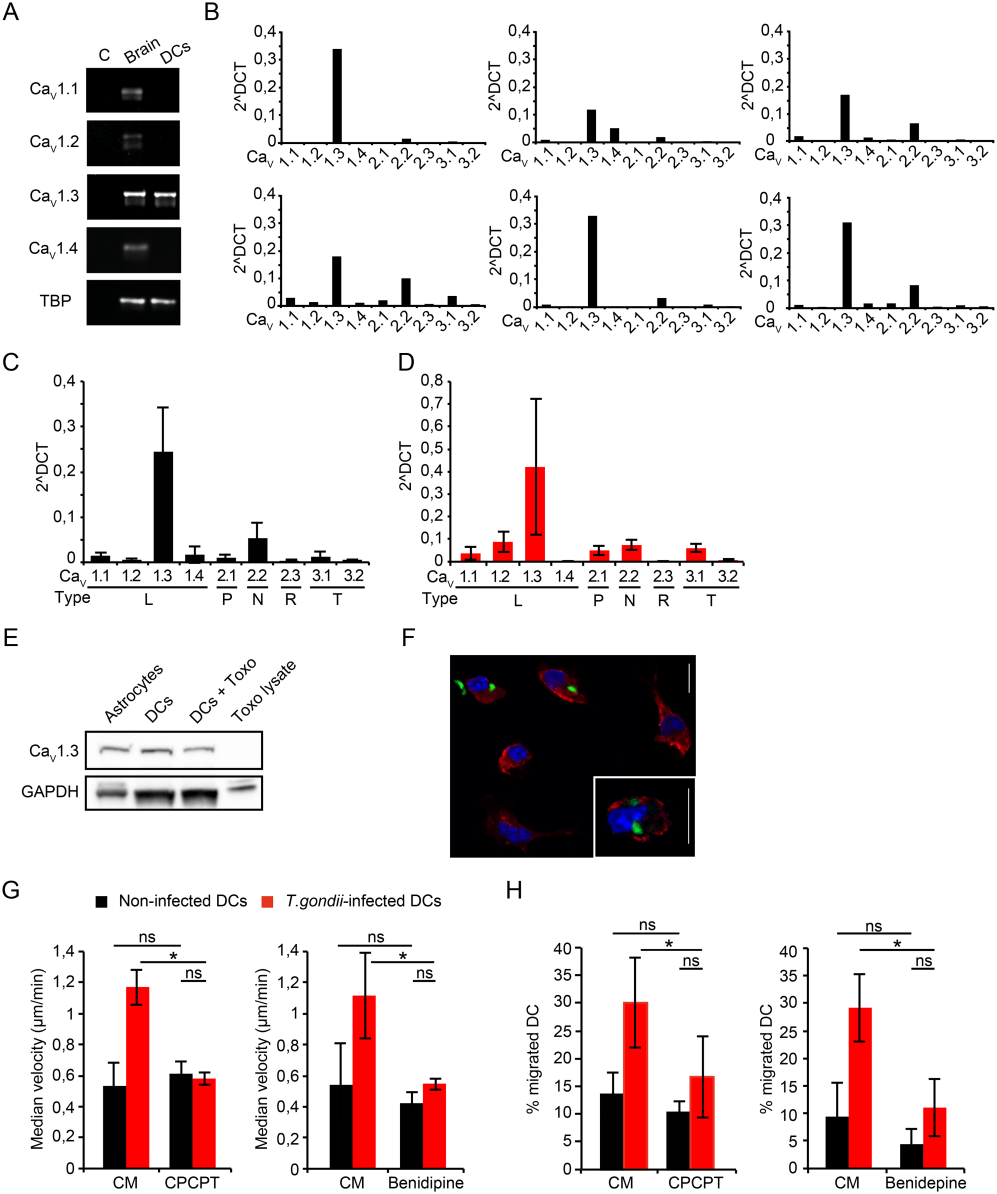
Expression of VDCCs in murine DCs and link to hypermigration of Toxoplasma-infected DCs. **(A)** RT-PCR using primers against Ca_V_ α_1_ subunits Ca_V_1.1, 1.2, 1.3, 1.4, as detailed in Materials and Methods. Gel shows template negative (C), whole brain lysate (brain) and DC cDNA. Data are representative of 3 independent experiments. **(B)** qPCR using primers against α_1_ subunits of Ca_V_1.1, 1.2, 1.3, 1.4, 2.1, 2.2, 2.3, 3.1 and 3.2 as detailed in Materials and Methods. Each graph depicts expression levels in DCs derived from one individual mouse (n = 6). ΔCt values were calculated with TBP as reference gene. (**C**) Compiled qPCR analysis as in (B). ΔCt values are given as means ± SEM of 6 independent experiments performed in duplicate. **(D)** Compiled qPCR analysis as in (C) for *T*. *gondii*-challenged DCs. ΔCt values are given as means ± SEM of 4 independent experiments performed in duplicate. **(E)** Western blot of lysates from primary astrocytes, unchallenged DCs (DCs), DCs challenged with *T*. *gondii* tachyzoites (DCs+ Toxo) and tachyzoite lysate (Toxo lysate) immunoblotted with Ca_V_1.3 mAb as detailed in Materials and Methods. GAPDH was used as loading reference. **(F)** Immunocytochemistry of DCs incubated with GFP-expressing *T*. *gondii* tachyzoites (green), stained with Ca_V_1.3 monoclonal antibody (red) and DAPI (blue) as described in Materials and Methods. Scale bars: 10 μm. **(G)** Motility analysis of DCs challenged with *T*. *gondii* tachyzoites (PTG, 3 h, MOI 3) followed by treatment (1 h) with selective Ca_V_1.3 inhibitor CPCPT (1 μM) or benidipine (10 μM), respectively. Data represent median velocities ± SD of 3 independent experiments. Asterisks indicate significant differences (*: p < 0.001, ns: p ≥ 0.05, Pairwise Wilcoxon rank-sum test, Holm correction). **(H)** Transmigration frequency of DCs challenged with *T*. *gondii* tachyzoites followed by treatment (1 h) with CPCPT (10 μM) or benidipine (40 μM), respectively. Data represent means ± SD of 3 independent experiments performed in duplicate. Asterisks indicate significant differences (*: p < 0.02, One-way ANOVA, Tukey’s HSD test).

In order to determine putative VDCCs mediating the nifedipine-sensitive GABA reconstitution effect, we performed a screen of VDCCs expressed in DCs. RT-PCR analyses indicated transcriptional expression of the L-type VDCC Ca_v_1.3 in DCs, similar to brain homogenate (Fig 5A). A real-time quantitative PCR (qPCR) screen of VDCCs confirmed a consistent high relative expression of Ca_v_1.3 transcripts in 6 mice tested over time, and also less abundant relative expression of Ca_v_2.2 (Fig 5B and 5C). Other VDCC types, e.g. Ca_v_1.1, Ca_v_1.4, Ca_v_2.1, Ca_v_3.1, exhibited low, undetectable or inconsistent relative expression (Fig 5B and 5C). In Toxoplasma-challenged DCs, Ca_v_1.3 remained the predominantly expressed VDCC type over other types (Fig 5D and S4A Fig) and maintained transcriptional expression of Ca_v_1.3 in Toxoplasma-infected DCs related to non-challenged DCs was observed during 24 h infection (S4B Fig). Western blot analyses detected polypeptides (≈ 250 kDa) in DCs, corresponding to Ca_v_1.3 expression as previously characterized in primary astrocytes [23], and with similar relative expression in DCs and Toxoplasma-infected DCs (Fig 5E and S4C Fig). Immunocytochemistry using a mAb to a predicted sub-membranous Ca_v_1.3 epitope yielded a distinct fluorescence signal in non-infected and in infected permeabilized DCs (Fig 5F). Altogether, we conclude that Ca_v_1.3 was the predominantly expressed VDCC in murine bone marrow-derived DCs and that the relative VDCC expression profile varied between mice or varied over time. Upon Toxoplasma-infection, Ca_v_1.3 remains the predominant transcriptionally expressed VDCC.

To functionally assess the relative contribution of Ca_v_1.3 to hypermigration in relation to other putatively expressed VDCCs, we took advantage of a pharmacological antagonist with high specificity for Ca_v_1.3, CPCPT [24], and a broad inhibitor of L, N and T type VDCCs, benidipine [25]. Both inhibitors similarly abolished the hypermotility of infected DCs (Fig 5G and S4D Fig). While CPCPT significantly reduced transmigration of DCs from different mice, benidipine was a more consistent abrogator of transmigration (Fig 5H). Jointly, these data suggest that VDCCs play a significant role in *T*. *gondii-*induced hypermotility of DCs. As Ca_v_1.3 appeared to be the most abundantly expressed VDCC, these data suggested that CPCPT and benidipine might act primarily on Ca_v_1.3.

### Ca_v_1.3 gene silencing abrogates the hypermotility of *T*. *gondii*-infected DCs

**Fig 6:**
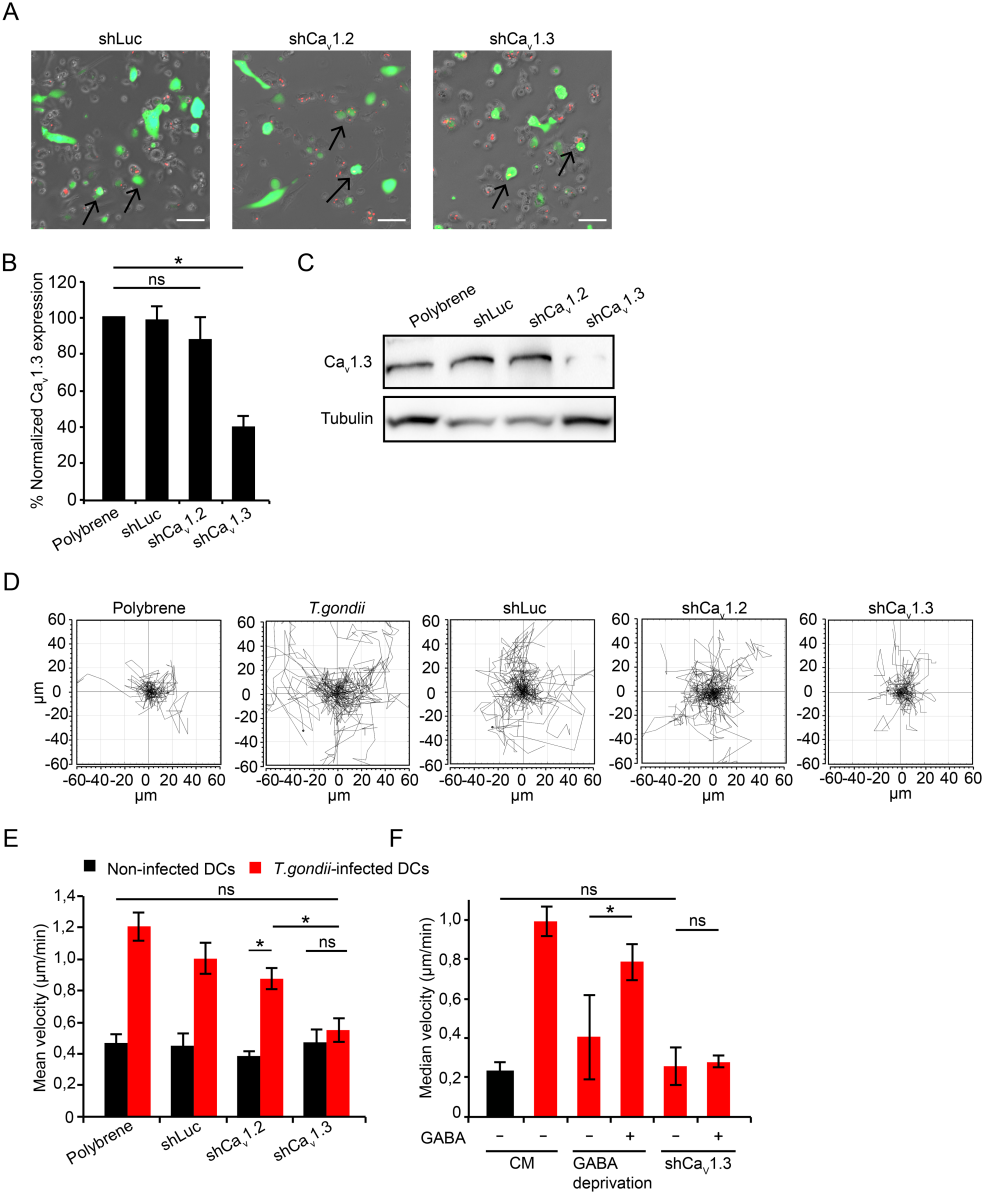
Ca_V_1.3 gene silencing abolishes DC hypermotility. **(A)** Live cell imaging of DCs transduced with EGFP-expressing lentiviral vectors (green) carrying shRNA targeting Ca_V_1.3 (shCa_v_1.3), Ca_V_1.2 (shCa_v_1.2) or a non-related target (Control shRNA, shLuc) as indicated under Materials and Methods. DCs were challenged with RFP-expressing tachyzoites (PRU, MOI 3, 4 h, red). Arrowheads indicate representative infected cells expressing EGFP-reporter (red + green) assessed in the assay. Scale bar: 50 μm. (**B**) Relative Ca_v_1.3 expression in DCs transduced with shCa_v_1.3, shCa_v_1.2 and control shLuc related to mock-transduced DCs (polybrene-treated). Data represents mean ± SEM from 4 independent experiments. (*: p < 0.05, ns: p ≥ 0.05, Student´s *t-*test). **(C)** Expression of Ca_v_1.3 protein after treatment with polybrene and transduction with shLuc, shCa_v_1.2 or shCa_v_1.3, analyzed by Western blotting as indicated under Materials and Methods. Data is representative of 3 independent experiments. (**D**) Motility plots of DCs transduced with lentiviral vectors carrying shRNA targeting Ca_V_1.2, Ca_V_1.3 or control shLuc and challenged with *T*. *gondii* tachyzoites (PRU, MOI 3) as indicated under Materials and Methods. “Polybrene” indicates mock-transduced DCs. Data is representative of 4 independent experiments. (**E**) Motility analysis of DCs transduced as in (D). Data represent mean velocities ± SD of 4 independent experiments. Asterisks indicate significant differences (*: p < 0.001, ns: p ≥ 0.05, Pairwise Wilcoxon rank-sum test, Holm correction). **(F)** Motility analysis of DCs transduced with recombinant lentiviral vectors carrying shRNA targeting Ca_v_1.3. DCs were challenged with *T*. *gondii* tachyzoites ± SC (50 μM, GABA-deprived) ± GABA (5 μM). CM indicates complete medium. Data represent median velocities ± SD of 2 independent experiments. Asterisks indicate significant differences (*: p < 0.01, ns: p ≥ 0.05, Pairwise Wilcoxon rank-sum test, Holm correction).

To test the functional implication of Ca_v_1.3 in *T*. *gondii*-induced hypermotility, we employed an RNA interference approach. First, transduction efficacy by the recombinant lentiviral vector was optimized in the murine neuroectodermal cell line NE-4C and in primary DCs (S5 Fig). Ca_v_1.3 (shCa_v_1.3) and Ca_v_1.2 (shCa_v_1.2) were successfully targeted in NE-4C cells by this approach (S6A, S6B and S6C Fig). Similarly, in primary DCs, shRNA targeting Ca_v_1.3 (shCa_v_1.3), Ca_v_1.2 (shCa_v_1.2) or control shRNA (shLuc) was delivered and the transduced DCs were challenged with *T*. *gondii* tachyzoites (Fig 6A). DCs transduced with shCa_v_1.3 exhibited significantly reduced Ca_v_1.3 mRNA expression, with non-significant effects on Ca_v_1.3 mRNA expression by shCa_v_1.2 and control shRNA (Fig 6B). Western blotting analyses of DCs and NE-4C cells transduced with shCa_v_1.3 showed a reduction in Ca_v_1.3 protein expression (Fig 6C, S6D Fig).

Because primary DCs may become activated by the lentivirus and activation may impact on motility, we assessed expression of IL-12 mRNA in primary DCs and the NE-4C line. While the expression of IL-12 mRNA was relatively unaffected in NE-4C cells, primary DCs exhibited enhanced expression of IL-12 mRNA upon lentiviral transduction, in a similar fashion for shLuc, shCa_v_1.2 and shCa_v_1.3 (S7 Fig). We conclude that Ca_v_1.3 mRNA and protein expression were selectively reduced in DCs exposed to shCa_v_1.3 and that lentiviral transduction generates enhanced IL-12 mRNA expression in primary DCs.

To assess the impact of Ca_v_1.3 silencing on hypermotility, we first optimized the approach using the murine DC line (JAWS II). JAWS II cells and DCs expressed a similar VDCC profile, with Ca_v_1.3 as the most prominently expressed VDCC (S8A Fig) and a similar inhibitory profile by calcium blockers on hypermotility was observed (S8B Fig). JAWS II transduced with shCa_v_1.3 (S8C Fig) exhibited significantly reduced Ca_v_1.3 mRNA expression and enhanced IL-12 mRNA expression (S8D and S8E Fig). Importantly, shCa_v_1.3-tranduced primary DCs (Fig 6D) and JAWS II exhibited reduced motility upon Toxoplasma-challenge. Their velocities reached non-significant differences compared with baseline motility of non-infected DCs (Fig 6E) and JAWS II, respectively (S8F Fig). Significant differences in the reduction of motility were observed for shCa_v_1.3-tranduced DCs compared with shCa_v_1.2-, shLuc- and mock-transduced DCs (Fig 6E). In line with results obtained upon pharmacological L-type VDCC inhibition (Fig 4D), exogenous GABA restored motility in mock-treated GABA-inhibited DCs but failed to restore motility in the shCa_v_1.3-transduced cells (Fig 6F). We conclude that selective silencing of Ca_v_1.3 abolishes *T*. *gondii*-induced hypermotility in DCs.

### VDCC inhibition in adoptively transferred infected DCs delays the dissemination of *T*. *gondii* in mice

**Fig 7:**
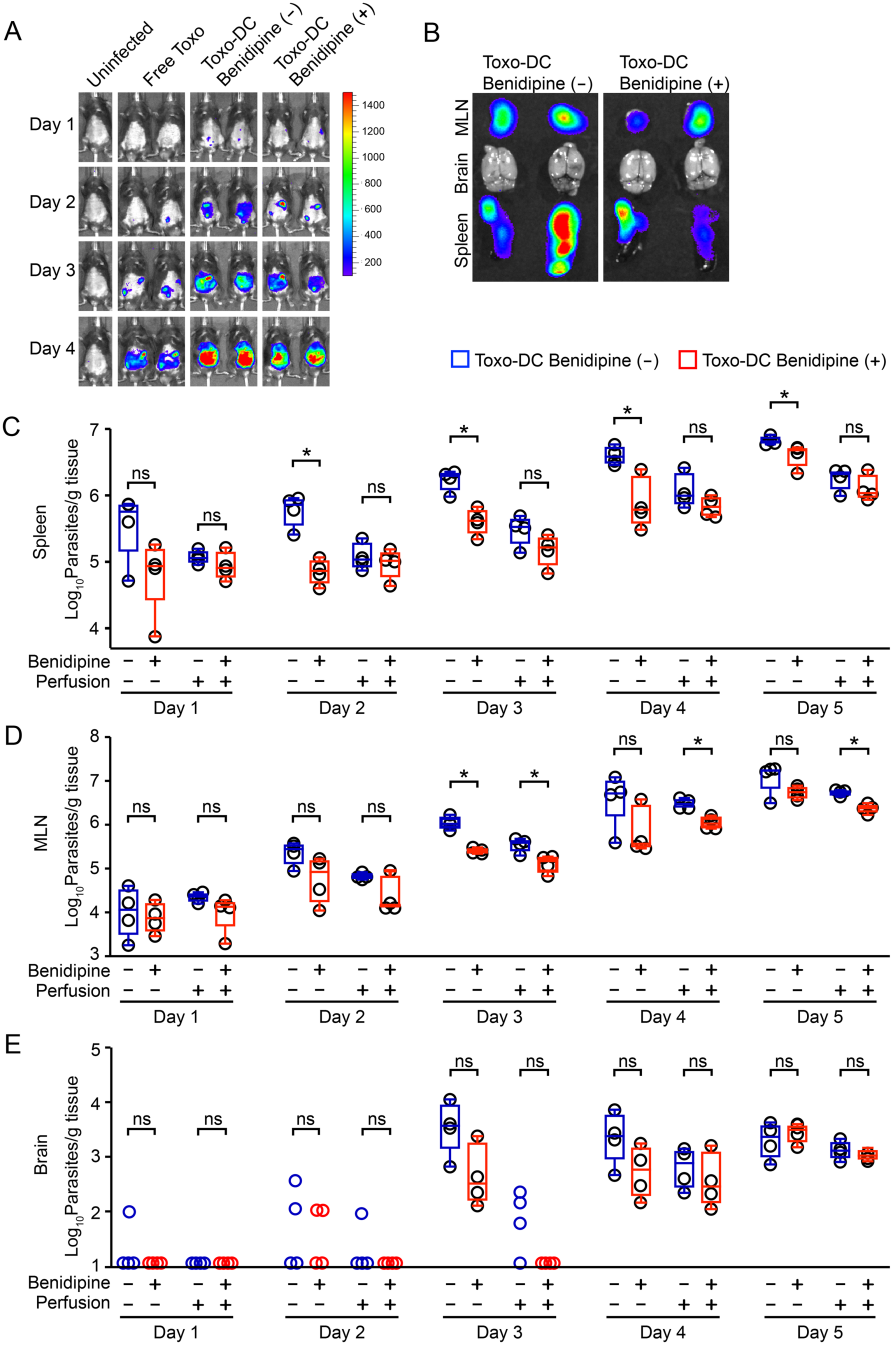
Parasite loads in non-perfused and perfused mice upon VDCC inhibition in adoptively transferred Toxoplasma-infected DCs. (**A**) C57BL/6 mice were challenged with 5x10^4^ cfu of freshly egressed PTGluc tachyzoites (Free Toxo), 5x10^4^ cfu of tachyzoite-challenged DCs (Toxo-DC Benidipine (-)) or 5x10^4^ cfu tachyzoite-challenged DCs treated with benidipine (Toxo-DC Benidipine (+)). Photonic emissions were assessed by BLI on days 1–4 post-inoculation intraperitoneally. Color scales indicate photon emission (photons/s/cm^2^/sr) during 180 s exposures. Data are representative of 2 mice from each group (n = 4/group). (**B**) *Ex vivo* photonic emissions from spleen, MLN and brains of mice as in (A) on day 5 post-inoculation. Representative data from 2 mice from each group are shown. Scale as in (A). (**C, D and E**) Parasite loads in spleen, MLN and brain, respectively, on days 1–5 post inoculation quantified by plaquing assays as indicated under Materials and Methods. C57BL/6 mice were inoculated intraperitoneally with 10^5^ cfu of tachyzoite (PTGluc)-challenged DCs ± benidipine pre-treatment, as indicated. Organ extraction was performed without blood perfusion or posterior to perfusion, as indicated. Open circles indicate individual mice. Box-plot and whiskers graphs represent the lower, upper quartiles and median (*: p < 0.05, ns: p ≥ 0.05, Student´s *t*-test, n = 4).

We have previously shown that adoptive transfer of *T*. *gondii*-infected DCs in mice leads to rapid dissemination of parasites and to exacerbation of the infection compared to infection with free tachyzoites [5, 26], and that GABAergic inhibition blocks this exacerbated dissemination [8]. To assess if VDCC inhibition impacted on parasite loads, benidipine pre-treated infected DCs were adoptively transferred to mice intraperitoneally. When the infections were monitored by *in vivo* bioluminescence, photonic emissions indicated dissemination of parasites to spleen and mesenteric lymph nodes (MLN) (Fig 7A and 7B). Plaquing assays of homogenized whole organs (non-perfused) revealed overall reduced mean parasite loads in the spleens of mice challenged with infected DCs (+) benidipine compared to mice challenged with infected DCs (-) benidipine (Fig 7C). To analyze the contribution of parasites in the blood circulation to the total parasite loads in organs, parasite loads were analyzed after blood perfusion. Perfused spleens exhibited overall reduced parasite loads, and reduced or abolished differences in parasite loads between the benidipine-treated and the non-treated conditions (Fig 7C). This showed that both removal of blood and benidipine treatment had a reducing impact on parasite loads in the spleen. In contrast, blood perfusion yielded more discrete relative reductions of parasite loads in MLNs, which are indirectly linked to the blood circulation via the lymphatic system (Fig 7D). In the perfused mice, parasites were consistently detected in all brains from day 4 versus day 3 in non-perfused mice (Fig 7E), indicating a contribution of parasites that were displaceable by blood perfusion to the total parasite loads in non-perfused mice.

**Fig 8:**
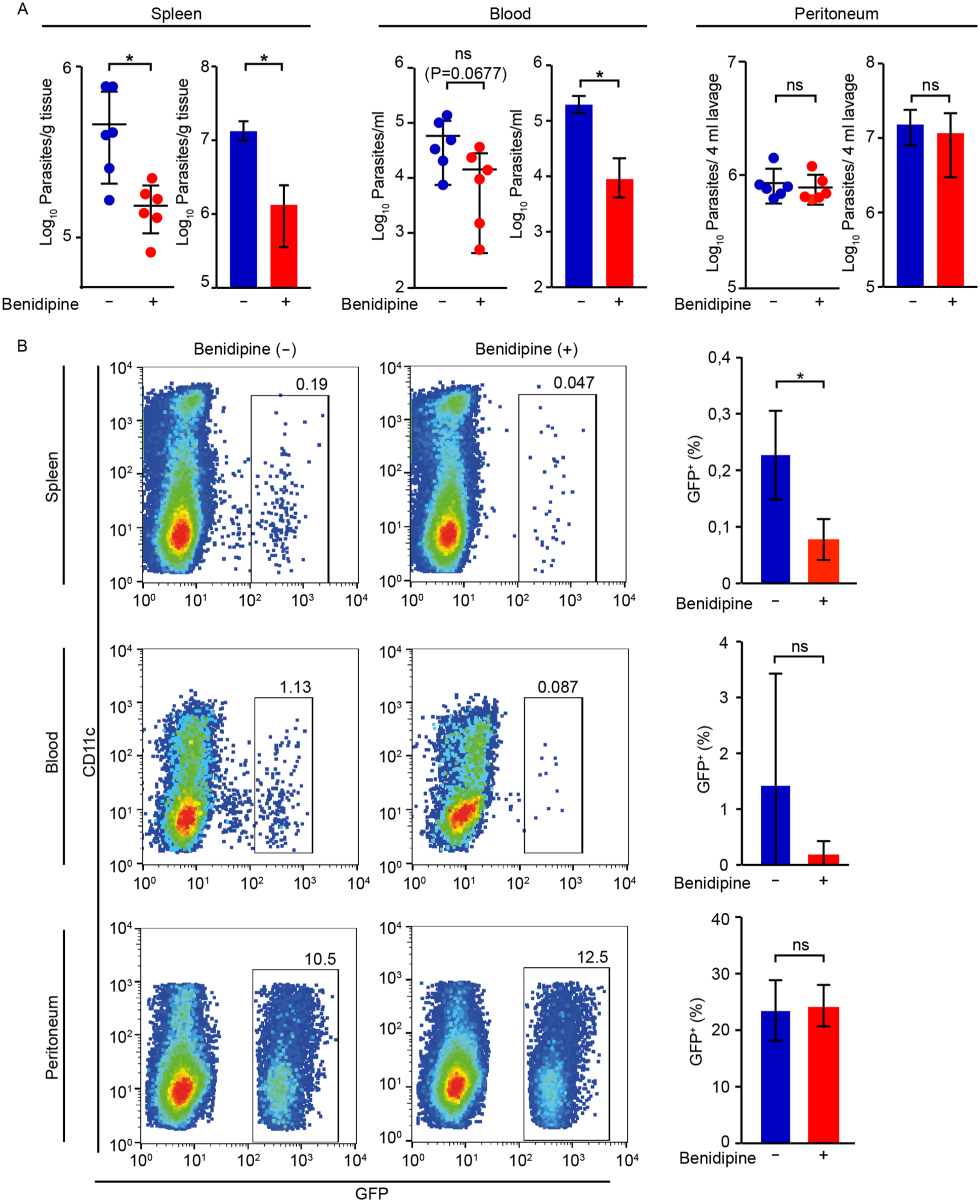
Impact of VDCC inhibition on parasite loads and cell-associated parasite numbers in spleen, blood and peritoneum 24 h post-inoculation in mice. **(A)** Parasite loads in spleen, blood and peritoneum, as indicated, 24 h post-inoculation of 5x10^6^ *T*. *gondii* (PTGluc)-challenged DCs ± benidipine in C57BL/6 mice. For each tissue, dot plots show parasite numbers quantified by plaquing assays and bar graphs show parasite quantification by qPCR, as indicated under Material and Methods. Data represents mean ± SD from 3 independent experiments. (*: p < 0.05, ns: p ≥ 0.05, Student´s *t*-test, n = 6). **(B)** Flow cytometry analysis of spleen, blood and peritoneal fluid 24 h post-inoculation of 5x10^6^ *T*. *gondii* (PTGluc, GFP-expressing)-challenged DCs ± benidipine, as indicated under Materials and Methods. Bivariate dot plots show, for each condition, GFP^+^ cells and CD11c^+^ cells, following gating on live CD3^-^ CD19^-^ GR1^-^NK1.1^-^ CD11b^+^ cells. Gatings indicate percentage of GFP^+^ cells related to the total cell population. Bar graphs indicate, for each condition, the percentage of GFP^+^ cells from 3 independent experiments (*: p < 0.05, ns: p ≥ 0.05, Student´s *t*-test, n = 5–6).

Because benidipine-treatment had an impact on splenic parasite loads early during infection and this effect appeared linked to parasites in blood, we analyzed the fate of parasites and DCs within 24 h post-inoculation intraperitoneally. Upon benidipine pre-treatment of infected DCs, significantly reduced parasite numbers were measured in spleen by 24 h (Fig 8A), with a non-significant reduction of parasites in blood and non-significant differences in peritoneum (Fig 8A). Similarly, flow cytometry analyses identified reduced numbers of cell-associated GFP-expressing parasites (GFP^+^) in the spleen upon benidipine treatment (Fig 8B and S9A Fig), in line with the observed differences by plaquing assays and qPCR (Fig 8A). When adoptively transferred infected DCs were pre-labeled with a cell dye (CMTMR), CMTMR^+^ GFP^+^ cells were detected in the spleens and also CMTMR^-^ GFP^+^ cells (S9B Fig). This indicated direct transport to the spleen by infected DCs and also rapid transfer of parasites to new leukocytes in peritoneum and spleen. Benidipine treatment yielded non-significant effects on DC viability, infection frequencies and parasite viability (S10 Fig). Altogether, the data show that adoptively transferred infected DCs rapidly entered the circulation and that VDCC inhibition led to reduced numbers of parasite-associated cells in spleen during the early phase of infection. VDCC inhibition delayed the appearance of parasites in circulation and, thereby, also the systemic dissemination of *T*. *gondii*.

## Discussion

In this study we investigated the molecular signaling mechanisms that govern how *T*. *gondii* hijacks the migratory properties of DCs. Building on previous work showing that a hypermigratory phenotype sets in within a few minutes after *T*. *gondii* invasion of DCs [4] and depends on GABAergic signaling [8], we addressed the role of Ca^2+^ signaling in these processes.

Our studies establish that Ca^2+^ signaling in murine bone marrow-derived DCs is indispensable for *T*. *gondii-*induced hypermotility and transmigration *in vitro*. The observation that the onset of the hypermigratory phenotype was abrogated at sub-physiological extracellular Ca^2+^ concentrations or by blocking plasma membrane Ca^2+^ channels underpinned a role for membrane-bound Ca^2+^ channels. However, Ca^2+^ mediates signal transduction to multiple cellular pathways. It was therefore crucial to determine its putative interaction with the GABAergic system of DCs. We previously showed that inhibition of GABA synthesis, GABA secretion or GABA_A_ receptor blockade in Toxoplasma-infected DCs abolishes hypermigration [8]. Here, we demonstrate that hypermotility and transmigration are restored in GABA-deprived infected DCs by (*i*) addition of exogenous GABA, (*ii*) by cell membrane depolarization with KCl and (*iii*) by L-type VDCC agonism. Consequently, (*iv*) L-type VDCC blockade hindered reconstitution of hypermotility by GABA and KCl. This pinpointed a role for VDCCs downstream of GABAergic signaling. Further, inhibition of purinergic Ca^2+^ channels (P2 receptors) yielded non-significant effects on hypermotility despite a measurable Ca^2+^ influx in response to ATP. Also, while Ca^2+^ -deprivation led to a similar proportional reduction of motility of unchallenged DCs (baseline motility) and infected DCs (hypermotility), selective L-type VDCC inhibition abolished hypermigration but had non-significant effects on the baseline motility of DCs. Altogether, this indicated that L-type VDCCs primarily mediated the GABA-evoked motility-related Ca^2+^ influx and that extracellular Ca^2+^ influx *per se* into the cell or increased cytosolic Ca^2+^ levels *per se* was not sufficient to induce hypermigration.

To our knowledge, the findings demonstrate for the first time that murine DCs express the L-type VDCC subtype Ca_v_1.3, with a functional implication in motility. Ca_v_1.3 appeared to be the predominant transcriptionally expressed VDCC in primary DCs, a feature also maintained by the DC line JAWS II. Importantly, silencing of Ca_v_1.3 by shRNA or selective pharmacological antagonism of Ca_v_1.3 abrogated the hypermigratory phenotype in Toxoplasma-infected DCs, while baseline motility and morphology of DCs remained intact related to mock-treated and non-infected DC. A caveat of lentiviral transduction in primary DCs is that the lentivirus vector may have activation effects on the DCs [27], yet without reported apparent inhibitory effects on functionality [28], thereof the requirement of appropriate control experiments. We validated and confirmed our results in two additional cell lines. IL-12 mRNA expression indicated activation by the lentiviral vector primarily in DCs, to a lesser extent in JAWS II and, non-significant effects on the NE-4C line. Silencing of Ca_v_1.3 expression abolished the hypermigratory phenotype, in contrast to Ca_v_1.2 silencing. This, together with its apparent predominant expression, attributes a primary role in Toxoplasma-induced hypermotility to the VDCC subtype Ca_v_1.3. However, despite that we did not observe compensatory up-regulation of other VDCCs/Ca_v_1.2 upon Ca_v_1.3 silencing in DCs, the data do not exclude a contributive role for other VDCC subtypes. In fact, the neuronal VDCC family members often display overlapping functions in mediating signal transduction [29]. This may apply to VDCCs in murine DCs too, as relative variations in transcription of several VDCC subtypes were detected in different mice over time, yet conserving a relative predominant expression of the subtype Ca_v_1.3. Altogether, the data at hand defines a role for Ca_v_1.3 in Toxoplasma-induced DC hypermotility and establish Ca^2+^ as a second messenger to GABAergic signaling in DCs.

We have previously shown that GABA induces GABA_A_ receptor-activated currents in DCs [8]. Here, we demonstrate that DCs can sense membrane voltage changes caused by depolarization (KCl or GABA) and can respond to GABA by a Ca^2+^ transient. The analogous hypermotility restoration effect of exogenous GABA and depolarization by KCl, together with the opposite effects of the VDCC inhibitors (benidipine, nifedipine, CPCPT) and the agonist BayK8644 (a structural analog of nifedipine with positive inotropic activity) strongly suggests that GABA mediates membrane depolarization-induced Ca^2+^ release via VDCCs. Also by analogy to findings in neurons [12], it is plausible that GABA_A_ receptors and chloride homeostasis are regulated by cation-chloride co-transporters in DCs. Altogether, our findings provide evidence of a direct link between GABA receptor signaling, Ca_v_1.3 activation and hypermotility.

Although modulated by Toxoplasma infection, functional GABA_A_ receptors appear to be constitutively expressed by murine and human DCs [8]. The effects of GABA on Ca^2+^ signaling via VDCCs / Ca_v_1.3 has not been previously addressed in immune cells [30]. However, Ca^2+^ channels mediate some of the most rapid biological processes described and VDCC signaling allows for immediate cellular responses to external stimuli [31]. This is in agreement with the features attributed to the hypermigratory phenotype of Toxoplasma-infected DCs [3]: for example, its rapid onset, cytoskeletal remodeling and switch to amoeboid-type of migration within minutes after *T*. *gondii* invasion of the host DC *in vitro* [4] and is also in line with the observed rapid migration of adoptively transferred DCs *in vivo* [8, 26].

It has been previously reported that VDCCs may play a role in DC maturation [18] and T cell activation [32]. VDCC-related activity on DCs has been implicated in engulfment of apoptotic bodies, IL-12-production and up-regulation of major histocompatibility complex II [17, 18], all of which are important immune functions of DCs. In line with these observations, the hypermigratory phenotype induced by *T*. *gondii* appears to rely on receptors and channels expressed by naïve DCs [8], but additionally requires the active invasion of a *T*. *gondii* tachyzoite [4], which is confirmed here by the observation that exogenous GABA *per se* is not motogenic on naïve DCs [8] (while VDCC agonism is). Altogether, this also advocates that *T*. *gondii* primes the host cell for responsiveness to GABA and is consistent with the idea that GABAergic activation occurs in an autocrine fashion with minimal by-stander effect [8]. Notably, the vast majority of GABA-responding DCs also responded to ATP or to consecutive stimuli with GABA ranging from micromolar to millimolar concentrations, indicating that GABA does not render the DCs refractory to other Ca^2+^-related stimuli and that intracellular Ca^2+^ homeostasis is rapidly restored. We cannot exclude the involvement of additional mechanisms for Ca^2+^ entry in DCs [33] acting sequentially or in parallel. However, their possible contribution to the hypermigratory phenotype should be secondary or posterior to Ca_v_1.3 activation, as silencing of Ca_v_1.3 in both primary DCs and the DC cell line JAWS II or selective pharmacological inhibition of Ca_v_1.3 [24] abrogated *T*. *gondii*-induced hypermotility. The posterior involvement of intracellular Ca^2+^ stores is also likely.

Ca^2+^ also controls a number of critical processes in apicomplexan parasites, including gliding motility, cell invasion and egress [34–36]. It is unlikely that these mechanisms play in the interpretation of our results as inhibitors were added posterior to parasite invasion and non-significant effects were observed on parasite viability, reinvasion after egress or after forced release from treated host cells. On the other hand, our observations suggest that, through activation of the GABAergic system, *T*. *gondii* modulates the Ca^2+^ homeostasis of the infected host cell, albeit transiently and locally. Induction of Ca^2+^ signaling offers the advantage of bypassing transcriptional regulation in the host cells and thereby accelerating effector functions, i.e. rapid migratory activation of the invaded DC and, thereby, dissemination. We have previously shown that the onset of *T*. *gondii*-induced hypermotility precedes chemotactic responses in DCs *in vitro* and that, after the onset of chemotaxis, GABA/GABA_A_ receptor-mediated hypermotility and CCR7-mediated chemotaxis can cooperatively enhance the migration of infected DCs *in vitro* [4, 8]. Thus, Ca^2+^ entry in DCs, secondary to GABAergic activation, could hypothetically also influence Ca^2+^-dependent chemotaxis, with propagation of the signal to intracellular Ca^2+^ stores. Future research needs to determine if Ca_v_1.3 is involved in the cytoskeletal rearrangements that accompany the onset of hypermotility, some of which are independent of GABAergic signaling [4], e.g. the dissolution of adhesion-related podosomes [4] and the switch to amoeboid-like high velocity migration [37].

Our data demonstrate that VDCC inhibition in adoptively transferred infected DCs delays the dissemination of *T*. *gondii* tachyzoites in mice. VDCC inhibition reduced the parasite numbers in circulation and in the spleen early after inoculation, likely by delaying the outmigration of infected DCs from the peritoneal cavity [38]. The data advocates that the early presence of parasites in blood is important for setting the parasite loads in mice and that VDCC inhibition delayed this process. In line with this, perfusion experiments showed that the circulating pool of parasites contributes to the total parasite loads in organs and to dissemination during acute infection. Also, the high variability in leukocyte-associated parasitemias between mice 24 h post-inoculation is in contrast with the lower variability of parasite loads in the organs later during infection, and may indicate that parasitemia is intermittent early after infection. Because the spleen is an early site of *T*. *gondii* replication during acute infection [39], this mobilizable pool of parasites (by blood perfusion) may be important for the systemic dissemination observed at later time points. Also, DCs and monocytic cells are parasitized early during infection [26, 38, 40] and, both leukocyte-associated tachyzoites [41] and extracellular (free) tachyzoites are detected in blood later during acute infection (day 4) [41]. Our data show that adoptively transferred infected DCs reach the circulation and spleen rapidly but also that the transfer of replicating tachyzoites to new leukocytes is rapid and can occur in the peritoneal cavity, in line with previous observations [42, 43]. Toxoplasma tachyzoites replicate in adoptively transferred DCs with lysis of infected DCs occurring within 48 h [5] and VDCC inhibition did not abrogate this process. This, together with the observed absence of parasites in brain parenchyma before day 4, indicates that it is unlikely that the adoptively transferred DCs transported parasites into the brain parenchyma. Rather, the observed delay in penetration to the parenchyma upon benidipine-treatment may be a consequence of delayed or lower parasitemias. Yet, DCs infiltrate the brain parenchyma during toxoplasmic encephalitis [44] and transportation of parasites to the brain by CD11b^+^ leukocytes has ben shown [40]. However, more recent findings show that replication of tachyzoites in the endothelium is necessary before passage to the brain parenchyma [41]. Our studies contribute to elucidating the role of infected DCs in circulation and their impact on systemic dissemination, which indirectly impacts on parasitic loads in the brain parenchyma, but do not specifically address the mechanisms of passage of *T*. *gondii* tachyzoites across the blood-brain barrier [8]. Jointly, mounting evidences show that Toxoplasma utilizes combined strategies for systemic dissemination [6], by hijacking leukocytes [5, 38, 40] and as free parasites [26, 41], and also with significant differences between Toxoplasma genotypes [26, 45]. Additionally, intracellular localization of tachyzoites in migratory leukocytes may offer a safe intracellular niche for replication and delivery to organs and vasculature.

**Fig 9:**
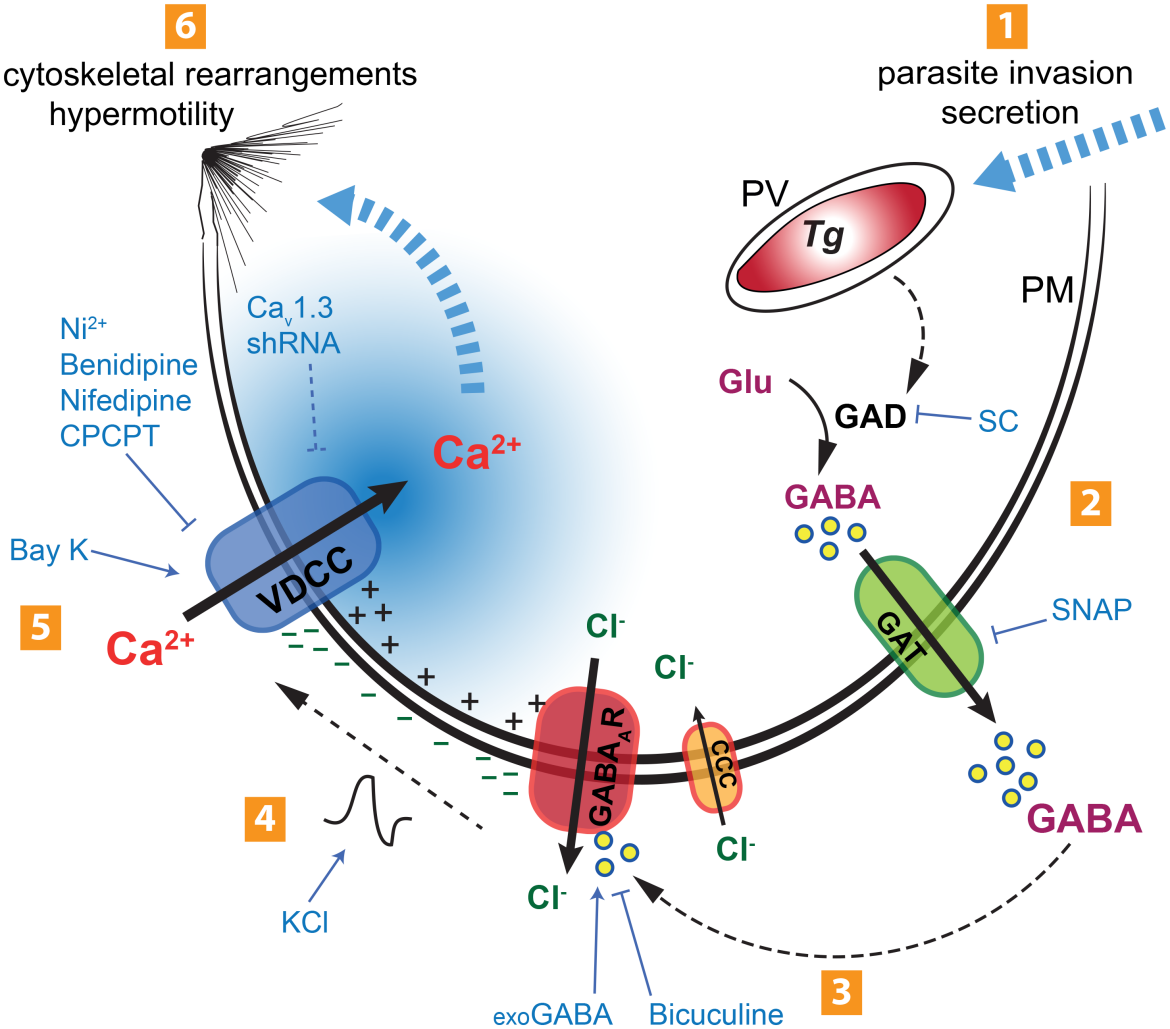
Schematic representation of the proposed mechanism for the initiation of the hypermigratory phenotype in Toxoplasma-infected DCs. (**1**) Active invasion by *T*. *gondii* (Tg) of the host cell across its plasma membrane (PM) involves secretory processes. Inside the host cell, *T*. *gondii* resides in the parasitophorous vacuole (PV). (**2**) Parasite invasion sparks an increase in GABA synthesis by host-cell glutamate decarboxylase (GAD) that can be inhibited by semicarbazide (SC) [8]. Synthesized GABA is secreted through GABA transporters (GAT). Inhibition of GABA synthesis (SC) or GABA secretion (SNAP) abolishes DC hypermotility [8]. (**3**) GABA activates GABA_A_ receptor (GABA_A_R) channels on the host cell surface by an autocrine loop, leading to chloride (Cl^-^) efflux. The GABA_A_R antagonist bicuculine inhibits hypermotility and exogenous GABA (exoGABA) reconstitutes hypermotility. Hypothetically, the Cl^-^ gradient is maintained by cation-chloride co-transporters (CCC). (**4**) Efflux of Cl^-^ leads to membrane depolarization (-). Induced membrane depolarization by potassium chloride (KCl) or GABA can also reconstitute hypermotility. (**5**) Voltage-dependent calcium channels (VDCCs; primarily Ca_v_1.3) open in response to membrane depolarization, leading to Ca^2+^ influx. Blockade of membrane-bound calcium channels by nickel (Ni), inhibition of VDCCs by nifedipine or benidipine, specific inhibition of Ca_v_1.3 (CPCPT) or ablation of Ca_V_1.3 expression by shRNA (Ca_V_1.3 shRNA) have inhibitory effects on hypermotility, while the VDCC agonist Bay K 8644 (Bay K) induces motility and reconstitutes hypermotility upon GABAergic inhibition. Upon Ca_v_1.3 blockade, GABA cannot induce or reconstitute hypermotility. (**6**) Hypothetically, the Ca^2+^ transient generated may be propagated to intracellular Ca^2+^ channels and stores. The altered Ca^2+^ signaling pattern may activate the downstream migratory machinery and MAP kinase regulators (e.g. 14-3-3), leading to cytoskeletal rearrangements and shifting the cell into a hypermotile state.

To the best of our knowledge, this constitutes the first report showing that the VDCC signaling axis can be utilized by an intracellular pathogen to modulate host cell migration and potentiate systemic dissemination. Based on the data at hand, we propose a model for the initiation of the hypermigratory phenotype in DCs by *T*. *gondii*, mediated by GABAergic signaling and with Ca^2+^ acting as a second messenger (Fig 9). Initially, tachyzoite invasion triggers activation of the GABAergic system—GABA synthesis, transport and activation of GABA_A_ receptors. Autocrine secretion of GABA by parasitized DCs leads to a membrane depolarization that activates the VDCC Ca_v_1.3, with entry of Ca^2+^ as a result. Finally, entry of Ca^2+^ activates downstream signaling pathways that lead to cytoskeletal rearrangements and hypermotility. Mounting evidence indicates that, rather than being passively transported, intracellular microorganisms induce refined molecular orchestrations to manipulate the signaling pathways that modulate the migration of infected immune cells [6, 46, 47]. Continued investigations into how intracellular pathogens manipulate host cell Ca^2+^ signaling pathways may identify new targets for inhibiting processes associated to pathogenesis.

## Materials and methods

### Ethics statement

The Regional Animal Research Ethical Board, Stockholm, Sweden, approved experimental procedures and protocols involving extraction of cells from mice (N135/15, N78/16), following proceedings described in EU legislation (Council Directive 2010/63/EU).

### Cells and parasites

Mouse bone marrow-derived DCs were generated and typified as previously described [8]. Briefly, cells from bone marrow of 6–10 week old C57BL/6 mice (Charles River) were cultivated in RPMI 1640 with 10% fetal bovine serum (FBS), gentamicin (20 μg/ml), glutamine (2 mM) and HEPES (0.01 M), referred to as complete medium (CM; all reagents from Life Technologies), and supplemented with 10 ng/ml recombinant mouse GM-CSF (Peprotech). Medium was replenished on days 2 and 4. Loosely adherent cells were harvested on day 6. The murine DC line JAWS II (CRL-11904) and murine neuroectodermal cell line NE-4C (CRL-2925) were cultured as indicated by the supplier (American Type Culture Collection). Primary astrocytes (ACs) were generated from cortices from 1–3 day-old C57BL/6 mice as previously described [48]. Freshly egressed *Toxoplasma gondii* tachyzoites of the RFP-expressing PRU-RFP [49] or GFP- and luciferase-expressing PTGluc [39] lines, kept on a 2-day passage cycle in murine fibroblast monolayers (L929, Sigma-Aldrich), were used in assays.

### Reagents

γ–aminobutyric acid (GABA), Adenosine triphosphate (ATP), (S)-SNAP-5114 (SNAP), semicarbazide (SC), nifedipine, Bay K8644, (all from Sigma-Aldrich), pyridoxalphosphate-6-azophenyl-2′,4′-disulfonic acid (PPADS), (4R)-rel-1,4-Dihydro-2,6-dimethyl-4-(3-nitrophenyl)-3,5-pyridinedicarboxylic acid 3-methyl 5-[(3R)-1-(phenylmethyl)-3-piperidinyl] ester hydrochloride (benidipine hydrochloride, all from Tocris) and 1-(3-Chlorophenethyl)-3-cyclopentylpyrimidine-2,4,6-(1H,3H,5H)-trione (CPCPT, Merck Millipore) were used at the indicated concentrations.

### Motility assays and transmigration assays

Motility assays were performed as previously described [4]. Briefly, 10^5^ DCs were incubated with freshly egressed tachyzoites (MOI 3, 4 h). The cells were mixed with collagen I (0.75 mg/ml, Life Technologies) and transferred to a chamber slide (Nalge Nunc Internat.) or 96-well plate. Imaging was performed for 1 h, 1 frame/min, at 100x magnification (Zeiss AxioImager). Time stacks were stabilized (Image Stabilizer, ImageJ) and motility data obtained by manual tracking of cells (Manual Tracking, ImageJ) of approximately 50–60 cells per condition. In infected samples, only cells where the RFP and DIC signals co-localized were tracked. Transmigration assays were performed as previously described [8]. Briefly, 10^6^ DCs were incubated with freshly egressed tachyzoites (MOI 3, 6 h), transferred into transwell filters (8 μm pore size; BD) in duplicate and incubated over night. Transmigrated DCs were quantified using a Neubauer hemocytometer. Ca^2+^-free medium was prepared from Ca^2+^-free DMEM, 1% FBS, gentamicin (20 μg/ml), glutamine (2 mM), 1 mM EGTA and HEPES (0.01 M), all reagents from Life Technologies.

### Ca^2+^ imaging

DCs (2x10^5^) were seeded on 5% 3-aminopropyltriethoxysilane coating glass bottom dish and incubated at 37°C with 5% CO_2_ for 15 min. DCs were then loaded with 2 μM Fluo-8H/AM (AAT Bioquest) in CM at 37°C with 5% CO_2_ for 15 min, and washed with Krebs-Ringer’s solution (150 mM NaCl, 6 mM KCl, 1.5 mM CaCl_2_, 1 mM MgCl_2_, 10 mM HEPES and 10 mM D-glucose) with 5% FBS. Time-lapse imaging was performed 2.5 s/frame, at 37°C with 5% CO_2_ on 200x magnification (Zeiss LSM 780 microscope equipped with a definite focus function). Cells were perfused with Krebs-Ringer’s solution with 5% FBS via a peristaltic pump (0.5 ml/min), which was also used to deliver pharmacological agents. The signals from individual cells were analyzed with ImageJ (version 1.46r, ROI Multi Measure). Each trace was normalized against the minimum value of all time points and a responding cell was defined as a signal exceeding 20% above baseline.

### Western blotting

To determine the expression of the Ca_V_1.3 protein in DCs and ACs, cells were lysed in RIPA buffer (150 mM NaCl, 50 mM Tris, 0.1% Triton, 0.5% deoxycholic acid, 0.1% SDS) with protease and phosphatase inhibitor cocktail (Thermo Fisher Scientific) followed by sonication, addition of 4 x laemmli sample buffer and boiling. Proteins were separated by 8% SDS-PAGE, and blotted onto PVDF membrane (Millipore), blocked in 2.5% BSA followed by Western blotting with monoclonal anti-Ca_V_1.3 C-terminal (Abcam), anti-GAPDH (Millipore) and anti-rabbit HRP (Cell signaling). Proteins were revealed by enhanced chemiluminescence (GE Healthcare) in a BioRad ChemiDoc XRS+.

### Immunocytochemistry

DCs (10^5^) were plated on poly-L-lysine coated coverslips and incubated with *T*. *gondii* tachyzoites (MOI 3, 4 h). Cells were fixed in 4% paraformaldehyde and permeabilized (0.1% Triton X-100), before incubation with mouse monoclonal anti-Ca_V_1.3 biotin (1:100, Abcam) and streptavidin-Alexa555 (1:500, Molecular Probes). Samples were treated with DAPI and imaged by confocal microscopy (Zeiss LSM780).

### Polymerase chain reaction (PCR)

Total RNA was extracted using TRIzol reagent (Life Technologies). First-strand cDNA was synthesized using Superscript III Reverse Transcriptase (Life Technologies). Real time quantitative PCR (qPCR) was performed in triplicates using SYBR green PCR master mix and a 7900HT Fast Real Time PCR system (Applied Biosystems). Products were analyzed with ABI 7900HT Sequence Detection System (Applied Biosystems) or Rotor gene (Corbett). 2^-ΔCt^ values are used to calculate the relative expression levels of 9 VDCC subtypes, with TATA box binding protein (TBP) as reference gene (S2 Table). For quantification of Ca_v_1.3 knock-down, glyceraldehyde 3-phosphate dehydrogenase (GAPDH) and actin were used as reference genes (S2 Table). For quantification of *T*. *gondii* in tissues, the B1 gene was used (S2 Table).

### Lentiviral vector production and transduction

Self-complementary hairpin DNA oligos targeting the Ca_V_1.2 (Cacna1c) mRNA, Ca_V_1.3 (Cacna1d) mRNA, and a non-related sequence (luciferase, Luc) were chemically synthesized (DNA Technology, Denmark), aligned and ligated in a self-inactivating lentiviral vector (pLL3.7) containing a CMV-driven EGFP reporter and a U6 promoter upstream of cloning restriction sites (HpaI and XhoI) [50] (S3 Table). Restriction enzyme analysis and direct DNA sequencing confirmed the correct insertion of short hairpin RNA (shRNA) sequences. Lentivirus production was done using lipofectamine transfection. Briefly, shCa_V_1.2, shCa_V_1.3 or shLuc vectors were co-transfected with psPAX2 packaging vector and pCMV-VSVg envelope vector into Lenti-X 293T cells (Clontech) and the resulting supernatant was harvested after 60 h. Recovered lentiviral particles were centrifuged to eliminate cell debris, filtered through 0.45-mm cellulose acetate filters and concentrated by ultracentrifugation. Titers were determined by infecting Lenti-X 293T cells with serial dilutions of concentrated lentivirus. NE-4C cells, JAWS II cells and DCs (day 3) were transduced by spinoculation at 1000 g for 30 min in presence of hexadimethrine bromide (Polybrene, 8 μg/ml; Sigma Aldrich). Three to 5 days post-transduction, EGFP-expression was verified by epifluorescence microscopy before the cells were used in experiments. Transduction frequency was defined as the number of EGFP-expressing cells related to the total numbers of cells in five representative fields of view.

### Mass spectrometry analysis

DCs (10^5^) were incubated with freshly egressed *T*. *gondii* PRU tachyzoites (MOI 3, 4 h). Cells were washed twice and incubated for 16 h in Krebs-Ringer’s solution supplemented with MEM essential and non-essential amino acids (Life Technologies) and 20 μg/ml gentamicin, referred to as *mod*. *R*. Inhibitors were present before and after the washes. 1 μL of cell supernatants were overlaid with 1μl of matrix (2.5 mg α-Cyano-4-hydroxycinnamic acid (HCCA) dissolved in 50% acetonitrile, 47.5% H_2_O, 2.5% TFA). Samples were analyzed by MALDI TOF mass spectrometry (Microflex LT, Bruker Daltronics) at laser frequency 60 Hz, mass range 0–1000 m/z, delayed ion extraction 100 ns, acceleration voltage 20 kV, lens voltage 6 kV and calibrated using the mass of HCCA matrix ions. Analysis was performed with flexAnalysis (version 3.3, Bruker Daltronics).

### Flow cytometry

Cells were collected from blood, peritoneum and spleen and depleted of red blood cells. Cells were then stained for CD11b (clone M1/70), CD11c (clone N418), CD19 (clone 1D3), NK1.1 (clone PK136), CD3 (clones 145.2C11) and live/dead marker Viability Dye eFluor 780 (eBioscience) or Fixable Yellow Dead Cell Kit (Invitrogen) following blocking of Fc receptors (24G2). All antibodies were from Biolegend (San Diego, CA). After 30 minutes incubation, the cells were washed extensively and then fixed prior to running on FACCyAN ADP LX 9-colour flow cytometer (Beckman Coulter, Pasadena, CA). Data were analyzed using FlowJo software (Tree Star Inc, OR).

### Adoptive transfers of Toxoplasma-infected DCs

Adoptive transfers were performed as previously described [8]. Briefly, DCs were challenged with freshly egressed PTGluc tachyzoites (6 h, MOI 3). Extracellular parasites were removed by centrifugation. Following resuspension in RPMI, tachyzoite-infected DCs or freshly egressed tachyzoites were adoptively transferred intraperitoneally into recipient C57BL/6 mice. Total number of colony-forming units (cfu) injected into animals was confirmed by plaquing assays. Benidipine (40 μM) was added to DCs for the last 3 h of the 6 h challenge with tachyzoites and replenished (40 μM) prior to injection in mice. When indicated, cells were stained with CMTMR following manufacturer´s instructions (Invitrogen).

### *In vivo* bioluminescence imaging (BLI)

Eight-10 week old C57BL/6 mice were inoculated i.p. with freshly egressed PTGluc tachyzoites, or with PTGluc-infected DC ± benidipine. 3 mg D-luciferin potassium salt (Caliper Life Sciences, Hopkinton, MA, USA) was injected i.p. and mice were anesthetized with 2.3% isoflurane prior to BLI. Ten min after injection of D-luciferin, biophotonic images were acquired for 180 s (medium binning) with an In Vivo Imaging System (Spectrum CT, Perkin Elmer). For *ex vivo* imaging, organs are extracted and assessed as above. Analysis of images and assessment of photons emitted from a region of interest (ROI) was performed with Live Imaging Software (version 4.2; Caliper Life Sciences).

### Plaquing assays

Plaquing assays were performed as described [8]. Briefly, organs were extracted and homogenized under conditions that did not affect parasite viability. The number of parasites was determined by plaque formation on fibroblast monolayers. When indicated, tachyzoites where released from infected DCs by repeated passages through a hypodermic needle (gauge 27), previous to plaquing. Transcardial blood perfusion was performed by injection of 25 ml PBS in the left ventricle after incision of the right atrium. Peritoneal lavage was performed by intraperinoneal perfusion and aspiration of 10 ml PBS using a hypodermic needle.

### Statistical analysis

Statistical analyses were performed using R Stats Package version 3.0.2 (R Foundation for Statistical Computing, Vienna, Austria). Normality was tested by the Shapiro-Wilks test. P-values > 0.05 were defined as non-significant.

## Supporting information

S1 Table Ca^2+^ response frequencies to GABA and ATP perfusion by DCs. (DOCX)

S2 Table Primers used to amplify VDCC subtype cDNA. (DOCX)

S3 Table Sequences of shRNAs. (DOCX)

S1 Fig Motility of unchallenged DCs under Ca^2+^ deprivation. **(A and B)** Histograms show distributions of accumulated distances migrated by unchallenged DCs in the presence (**A**) or absence (**B**) of extracellular Ca^2+^. Vertical red lines indicate, for each condition, the median distance migrated by cells. Significant differences in distances migrated were observed between the conditions (p < 0.001, Wilcoxon rank-sum test, Holm correction). Data are representative of 3 independent experiments.
(TIF)

S2 Fig Sequential calcium responses of DCs to GABA. **(A)** Relative fluorescence intensity of DCs loaded with 2 μM Fluo-8H/AM as described in Materials and Methods. Bars indicate perfusions of 100 μM GABA (2.5–3.5 min), 1 mM GABA (5–6 min), 10 mM GABA (7.5–8.5 min), and 50 μM ATP (10–11 min), respectively. Displayed data are representative traces from 3 independent experiments. **(B)** Bar graph shows percentage of cells responding to GABA at the indicated concentrations. Data represent mean ± SD from 3 independent experiments.
(TIF)

S3 Fig Mass spectrometry analysis of chemical grade GABA analytical standard. Mass spectrometry analysis of GABA chemical grade analytical standard dissolved in modified Krebs-Ringer’s solution as indicated under Materials and Methods. Data are representative of 3 independent experiments.
(TIF)

S4 Fig VDCC expression profile of Toxoplasma-infected DCs and effect on DC motility by Ca_v_1.3 inhibition (CPCPT) and by VDCC inhibition (benidipine). (**A)** qPCR using primers against α_1_ subunits of Ca_V_1.1, 1.2, 1.3, 1.4, 2.1, 2.2, 2.3, 3.1 and 3.2 as detailed in Materials and Methods. Each graph depicts expression levels in DCs derived from one individual mouse (n = 4). ΔCt values were calculated with TBP as reference gene. **(B)** Ca_V_1.3 expression in *T*. *gondii*-infected DCs. qPCR analysis of cDNA from DCs challenged with *T*. *gondii* tachyzoites (PRU, MOI 3) related to DCs in complete medium at indicated time-points and using primers against the α_1_ subunit of Ca_V_1.3 as detailed in Materials and Methods. ΔCt values were calculated with TBP as reference gene and are given as means ± SEM of 3 independent experiments performed in triplicate. (**C)** Ratiometric analysis of Ca_v_1.3 polypeptide expression by western blotting. Bar graph shows, for each condition, the relative expression (mean ± SD) after normalization to internal loading control (GAPDH) from 3 independent experiments (ns: p ≥ 0.05, Student´s *t*-test). **(D)** Representative motility plot analysis of DCs in complete medium (CM) or challenged with *T*. *gondii* tachyzoites (PTG, 3 h, MOI 3) followed by treatment (1 h) with the selective Ca_V_1.3 inhibitor CPCPT or benidipine, respectively. Data are representative of 3 independent experiments.
(TIF)

S5 Fig Transduction efficiency in the NE-4C cell line and in primary murine DCs. **(A and B)** Live cell imaging of DCs transduced with EGFP-expressing lentiviral vectors (green) carrying shRNA targeting Ca_V_1.3 (shCa_v_1.3), Ca_V_1.2 (shCa_v_1.2) or a non-related target (Control shRNA, shLuc). Tranductions were performed and evaluated by epifluorescence and light microscopy as indicated under Materials and Methods. The transduction frequencies of cells used in assays were consistently > 50% for primary DCs **(A)** and > 80% for NE-4C **(B)**. Data are representative of multiple independent transductions. Scale bar: 100 μm.
(TIF)

S6 Fig Validation of the gene silencing approach using the NE-4C cell line. **(A)** VDCC/Ca_v_1.3expression by the NE-4C line. Compiled qPCR analysis using primers against α_1_ subunits of Ca_V_1.1, 1.2, 1.3 and 1.4 as detailed in Materials and Methods. ΔCt values are given as means ± SEM of 3 independent experiments performed in duplicate. (**B**) Relative Ca_v_1.3 expression in NE-4C cells transduced with shCa_v_1.3, shCa_v_1.2 and control shRNA, shLuc related to polybrene-treated DCs assessed by qPCR as indicated under materials and Methods. Data represents means ± SEM of 3 independent experiments. (**C**) Relative Ca_v_1.2 expression in NE-4C cells transduced with shCa_v_1.2, shCa_v_1.3 and control shRNA, shLuc related to polybrene-treated DCs assessed by qPCR as indicated under materials and Methods. Data represents means ± SEM of 3 independent experiments. **(D)** Expression of Ca_v_1.3 protein after treatment with polybrene, transduction with shLuc, shCa_v_1.2 and shCa_v_1.3 analyzed by Western blotting as indicated under Materials and Methods. Data is representative of 3 independent experiments.
(TIF)

S7 Fig Expression of IL-12p35 mRNA in NE-4C and primary DCs upon lentiviral transduction. Relative IL-12 mRNA expression in **(A)** NE-4C cells and **(B)** primary DCs, respectively, following transduction with shLuc (control), shCa_v_1.2 or shCa_v_1.3 as indicated under Materials and Methods. Data shows fold increased expression related to polybrene treatment (1) and represents means ± SEM of 3 independent experiments performed in duplicate.
(TIF)

S8 Fig Characterization of the DC cell line JAWS II and effects of Ca_v_1.3 gene silencing on the motility of JAWS II upon *T*. *gondii* infection. **(A)** VDCC expression by the JAWS II line. Compiled qPCR analysis using primers against α_1_ subunits of Ca_V_1.1, 1.2, 1.3, 1.4, 2.1, 2.2, 2.3, 3.1 and 3.2 as detailed in Materials and Methods. ΔCt values are given as means ± SEM of 3 independent experiments performed in duplicate. **(B)** Motility analysis of JAWS II cells challenged with *T*. *gondii* tachyzoites (PTG, 3 h, MOI 3) followed by treatment (1 h) with nifedipine (10 μM), benidipine (10 μM) selective Ca_V_1.3 inhibitor CPCPT (1 μM) or purinergic calcium receptor inhibitor PPADS (100 μM). Data represent median velocities ± SD of 3 independent experiments. Asterisks indicate significant differences (*: p < 0.001, ns: p ≥ 0.05, Pairwise Wilcoxon rank-sum test, Holm correction). **(C)** Live cell imaging of JAWS II transduced with EGFP-expressing lentiviral vectors (green) carrying shRNA targeting Ca_V_1.3 (shCa_v_1.3) or a non-related target (Control shRNA, shLuc). Tranductions were performed and evaluated by epifluorescence and light microscopy as indicated under Materials and Methods. The transduction frequencies of cells were > 80% for JAWS II. Data are representative of multiple independent transductions. Scale bar: 100 μm. **(D)** Bar graph shows the relative Ca_v_1.3 expression in JAWS II cells transduced with shCa_v_1.3 and control shRNA (shLuc) related to polybrene-treated DCs assessed by qPCR as indicated under materials and Methods. ata represents means ± SEM of 3 independent experiments. **(E)** Bar graph (right) shows the relative IL-12 mRNA expression in JAWS II following transduction with shLuc (control) or shCa_v_1.3 as indicated under Materials and Methods. Data shows fold increased expression related to polybrene treatment (1). Data represents means ± SEM of 3 independent experiments. **(F)** Motility analysis of JAWS II transduced with recombinant lentiviral vectors carrying shRNA targeting Ca_v_1.3 or a non-related target (shLuc). DCs were challenged with *T*. *gondii* tachyzoites (PRU, MOI 3). Data represent median velocities ± SD of 3 independent experiments. Asterisks indicate significant differences (*: p < 0.001, ns: p ≥ 0.05, Pairwise Wilcoxon rank-sum test, Holm correction).
(TIF)

S9 Fig Characterization of DCs and fate of adoptively transferred Toxoplasma-infected DCs. **(A)** Flow cytometry analysis show the expression of CD11c and CD11b by murine bone-marrow-derived DCs utilized in adoptive transfers, as indicated under Materials and Methods. Plot is representative of multiple independent analyses. **(B)** Flow cytometry analysis of spleen and peritoneal fluid 24 h post-inoculation of 5x10^6^ *T*. *gondii* (GFP-expressing PTGluc)-infected DCs ± benidipine, as indicated under Materials and Methods. Cells were stained with CMTMR previous to inoculation. Representative bivariate dot plots show, for each condition, adoptively transferred parasite-associated DCs (CMTMR^+^ GFP^+^) and CMTMR^-^ parasite-associated cells (CMTMR^-^ GFP^+^). Analysis was gated on live CD3^-^ CD19^-^ GR1^-^NK1.1^-^ cells. Gatings indicate percentage of CMTMR^+^ GFP^+^ cells related to the total cell population. Plots are representative from 3 independent experiments. Bar graphs indicate, for each condition, the percentage of CMTMR^+^ GFP^+^ cells from 3 independent experiments (*: p < 0.05; ns: p ≥ 0.05, Student´s *t*-test, n = 3).
(TIF)

S10 Fig Effects of benidipine treatment on DC and *T*. *gondii* viability *in vitro* and *in vivo*. **(A)** Flow cytometry analysis of DCs challenged with GFP-expressing PTGluc tachyzoites in absence or presence of benidipine as indicated under Materials and Methods. Representative plots show infected DCs (GFP^+^) and viability (DCM^+^). **(B)** Bar graph shows percentage of DCM^+^ cells in presence or absence of benidipine as in (A). Data represent mean ± SD of 3 independent experiments (ns: p ≥ 0.05, Student´s *t*-test). (**C)** Plaquing assays of *T*. *gondii*-challenged DCs ± benidipine (40μM), nifedipine (30μM) or CPCPT (10μM). Following challenge with freshly egressed tachyzoites (PTGluc, MOI 3, 6 h) and treatment (3 h post-challenge), DCs were force-lyzed before plaquing as indicated under Materials and Methods. Equal volumes of force-lyzed challenged DC suspensions were plated and plaques were counted by epifluorescence microscopy. Bar graph shows compiled analyses of plaque counts related to the force-lyzed and mock-treated controls with complete medium (plaque counts in the mock-treated control was set to 1). Data represent mean ± SD of 3 independent experiments (ns: p ≥ 0.05, Student´s *t-*test).
(TIF)

S1 Movie DCs respond to GABA and ATP with a transient cytosolic Ca^2+^ increase. Live cell Ca^2+^ imaging of DCs loaded with 3 μM Fluo-4/AM as described in Materials and Methods. GABA (10 mM) and ATP (50 μM) were perfused after 3 min and 6.5 min, respectively. Data are representative of 3 independent experiments.
(MOV)
