# Supplementary material for: Voltage-dependent calcium channel signaling mediates GABAA receptor-induced migratory activation of dendritic cells infected by Toxoplasma gondii
Source: PLoS Pathog. 2017 Dec 7;13(12):e1006739. doi: 10.1371/journal.ppat.1006739 (PMC5720541; doi:10.1371/journal.ppat.1006739)
Supplement: S1 Table — (DOCX) [file ppat.1006739.s001.docx]

**Supplementary material**

**S1 Table. Ca^2+^ response frequencies to GABA and ATP perfusion by DCs.**

| Experiment | Cells responding to GABA^a^ | Cells responding to ATP ^b^ | Cells responding to GABA and ATP ^c^ | Total cells analyzed |
| --- | --- | --- | --- | --- |
| 1 | 3 (20%) | 7 (46.7%) | 3 (20%) | 15 |
| 2 | 13 (28.8%) | 12 (26.6%) | 9 (20%) | 45 |
| 3 | 10 (12.5%) | 44 (55%) | 10 (12.5%) | 80 |

DCs preloaded with Fluo-8H/AM were perfused with GABA and ATP, and the Ca^2+^ responses were assessed by microscopy as indicated under Materials and Methods.

^a^ Number of cells responding to GABA and frequency of responding cells (%) related to the total number of cells analyzed per experiment

^b^ Number of cells responding to ATP, analyzed as in ^a^

^c^ Number of cells responding to both GABA and ATP, analyzed as in ^a^
