# Supplementary material for: Voltage-dependent calcium channel signaling mediates GABAA receptor-induced migratory activation of dendritic cells infected by Toxoplasma gondii
Source: PLoS Pathog. 2017 Dec 7;13(12):e1006739. doi: 10.1371/journal.ppat.1006739 (PMC5720541; doi:10.1371/journal.ppat.1006739)
Supplement: S2 Table — (DOCX) [file ppat.1006739.s002.docx]

**S2 Table. Primers used to amplify VDCC subtype cDNA**

| Target | Primer pair sequence (5´to 3´) | Tm (°C) |
| --- | --- | --- |
| Cav1.2 | F: CGTTCTCATCCTGCTCAACA  R: TATGCTCCCAATGACGATGA | 60  60 |
| Cav1.3 | F: TGCACAGATGAAGCCAAAAG  R: GACCAACGTTCTCACCGTTT | 60  60 |
| Cav1.4 | F: TCCATCATGAAGGCGCTTGT  R: CGAGTCCGATGATGGCGTAA | 60  60 |
| Cav1.1 | F: GGTAGCATGTAAGAGGCTG  R: GCAAATAGTGTGGCATTAAAGG | 55  55 |
| Cav3.1 | F: TGTGGAAATGGTGGTGAAGA  R: ACTGCGGAGAAGCTGACATT | 60  60 |
| Cav3.2 | F: TGGGAACGTGCTTCTTCTCT  R: GGGGATGTGTGAGCATTTCT | 60  60 |
| Cav2.1 | F: AATTCCAAATCACGGAGCAC  R: CATCAGAAACGAGCACAGGA | 60  60 |
| Cav2.2 | F: GCAACACATGGAACTGGTTG  R: GCATTCTTGTCCTCCTCTGC | 60  60 |
| Cav2.3 | F: TGAAGGCTGTGTTTGACTGC  R: ATTCATGACGCTTCCATTCC | 60  60 |
| TBP | F: GGGGAGCTGTGATGTGAAGT  R: CCAGGAAATAATTCTGGCTCA | 60  60 |
| GAPDH | F: TGACCTCAACTACATGGTCTACA  R: CTTCCCATTCTCGGCCTTG | 59  58 |
| ACTIN  IL-12 p35  B1 | F: CACTGTCGAGTCGCGTCC  R: TCATCCATGGCGAACTGGTG  F: CCCTTGCCCTCCTAAACCA  R: CTAAGACACCTGGCAGGTCCA  F: GCATTGCCCGTCCAAACT  R: AGACTGTACGGAATGGAGACGAA | 60  60  59  61  58  61 |
|  |  |  |
|  |  |  |
|  |  |  |
|  |  |  |
|  |  |  |
|  |  |  |
