# Supplementary material for: Voltage-dependent calcium channel signaling mediates GABAA receptor-induced migratory activation of dendritic cells infected by Toxoplasma gondii
Source: PLoS Pathog. 2017 Dec 7;13(12):e1006739. doi: 10.1371/journal.ppat.1006739 (PMC5720541; doi:10.1371/journal.ppat.1006739)
Supplement: S3 Table — (DOCX) [file ppat.1006739.s003.docx]

**S3 Table. Sequences for shRNAs.**

| **Target** |  | **Sequence (5’ to 3’)** |
| --- | --- | --- |
| Control/  Luc |  | F: TGTTCTCCGAACGTGTCACGTTTCAAGAGAACGTGACACGTTCGGAGAACTTTTTTC |
| Cav1.2 |  | R: CGAGAAAAAAGTTCTCCGAACGTGTCACGTTCTCTTGAAACGTGACACGTTCGGAGAACA  F:  TGGCACCAGTACAAAGTGTGGTATTCAAGAGATACCACACTTTGTACTGGTGC-CTTTTTTC  R:  TCGAGAAAAAAGGCACCAGTACAAAGTGTGGTATCTCTTGAATACCACACTTTGTACTGGTGCCA |
| Ca_V_1.3 |  | F: TGGTAGGATTGTTTAGTGTAATTCAAGAGATTACACTAAACAATCCTACCTTTTTTC |
|  |  | R: TCGAGAAAAAAGGTAGGATTGTTTAGTGTAATCTCTTGAATTACACTAAACAATCCTACCA |
