# Supplementary figures and images for: Voltage-dependent calcium channel signaling mediates GABAA receptor-induced migratory activation of dendritic cells infected by Toxoplasma gondii
Source: PLoS Pathog. 2017 Dec 7;13(12):e1006739. doi: 10.1371/journal.ppat.1006739 (PMC5720541; doi:10.1371/journal.ppat.1006739)

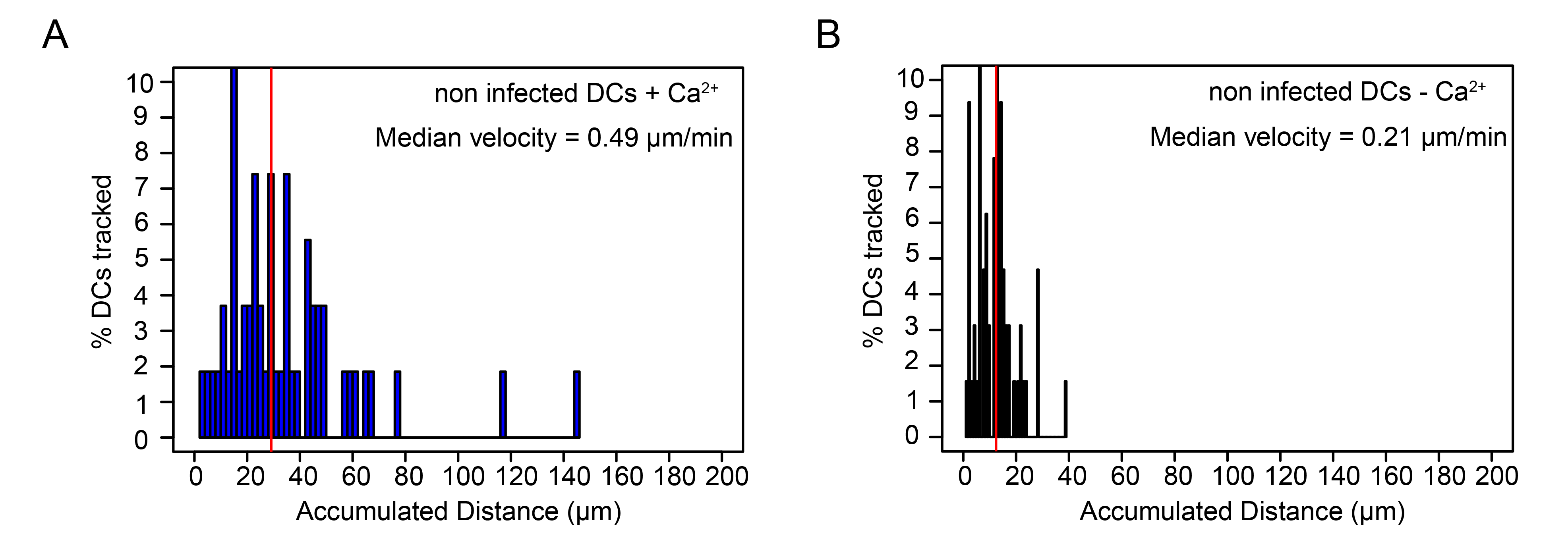

Supplement: S1 Fig — (A and B) Histograms show distributions of accumulated distances migrated by unchallenged DCs in the presence (A) or absence (B) of extracellular Ca2+. Vertical red lines indicate, for each condition, the median distance migrated by cells. Significant differences in distances migrated were observed between the conditions (p < 0.001, Wilcoxon rank-sum test, Holm correction). Data are representative of 3 independent experiments. (TIF) [file ppat.1006739.s004.tif]

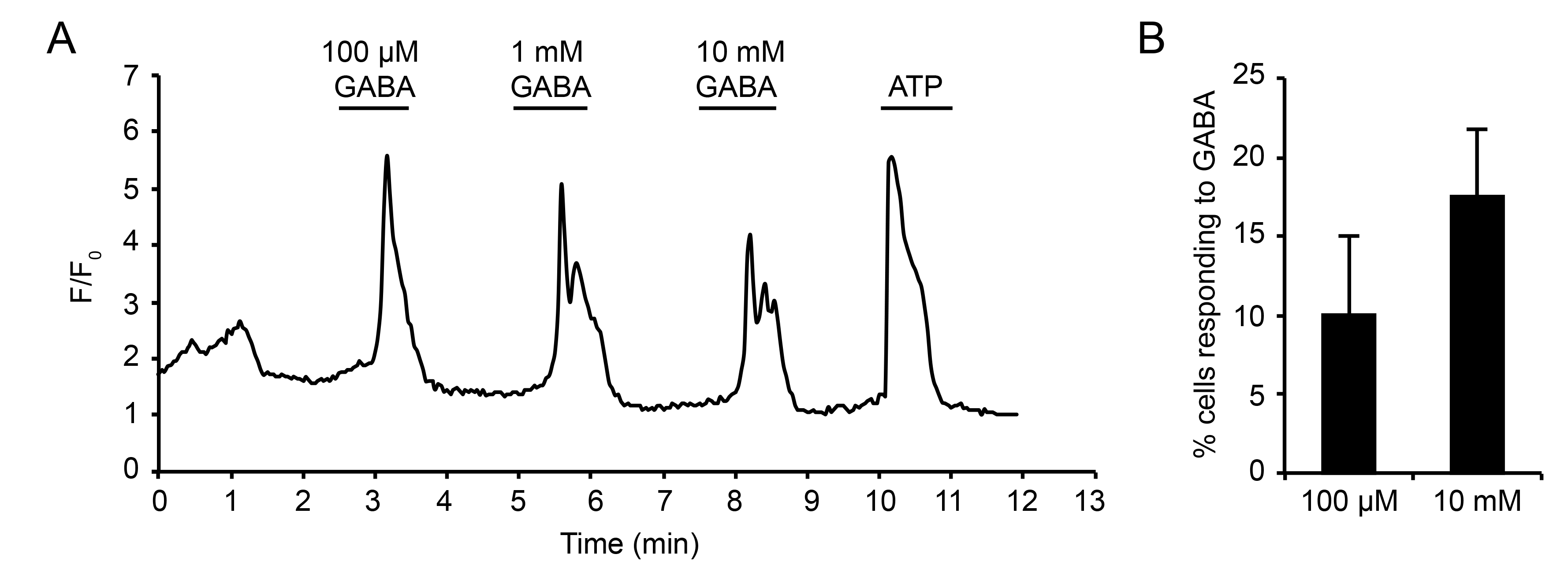

Supplement: S2 Fig — (A) Relative fluorescence intensity of DCs loaded with 2 μM Fluo-8H/AM as described in Materials and Methods. Bars indicate perfusions of 100 μM GABA (2.5–3.5 min), 1 mM GABA (5–6 min), 10 mM GABA (7.5–8.5 min), and 50 μM ATP (10–11 min), respectively. Displayed data are representative traces from 3 independent experiments. (B) Bar graph shows percentage of cells responding to GABA at the indicated concentrations. Data represent mean ± SD from 3 independent experiments. (TIF) [file ppat.1006739.s005.tif]

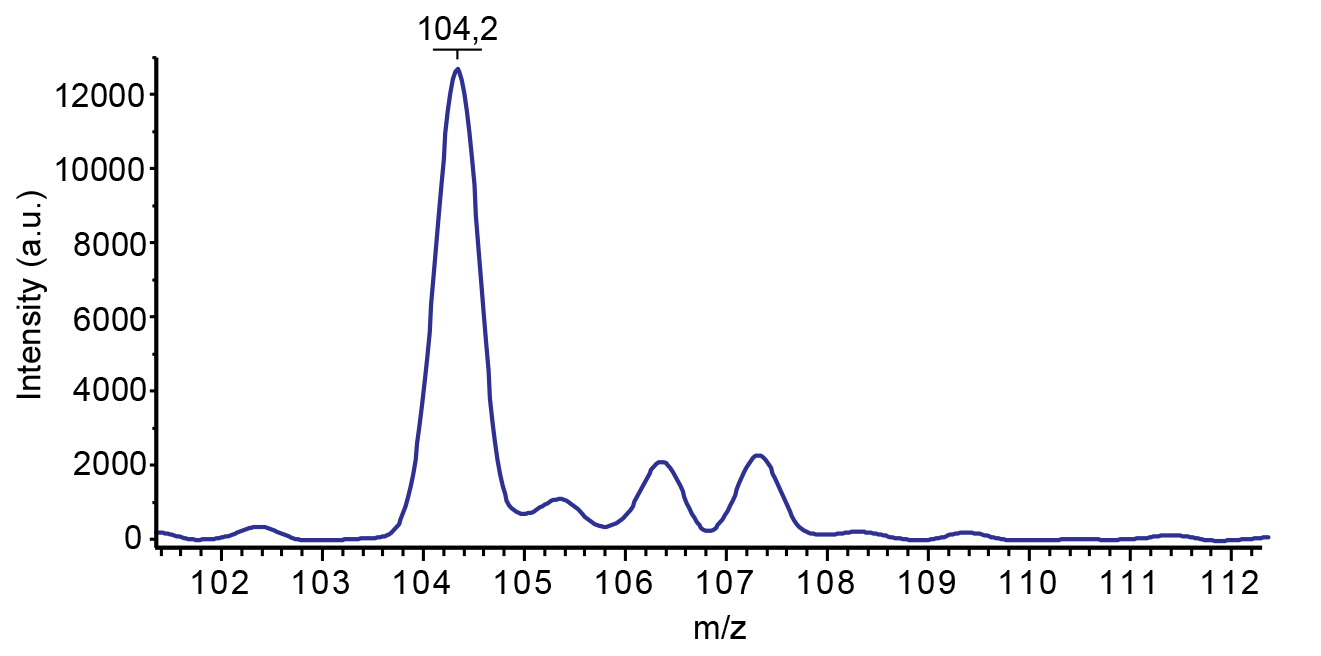

Supplement: S3 Fig — Mass spectrometry analysis of GABA chemical grade analytical standard dissolved in modified Krebs-Ringer’s solution as indicated under Materials and Methods. Data are representative of 3 independent experiments. (TIF) [file ppat.1006739.s006.tif]

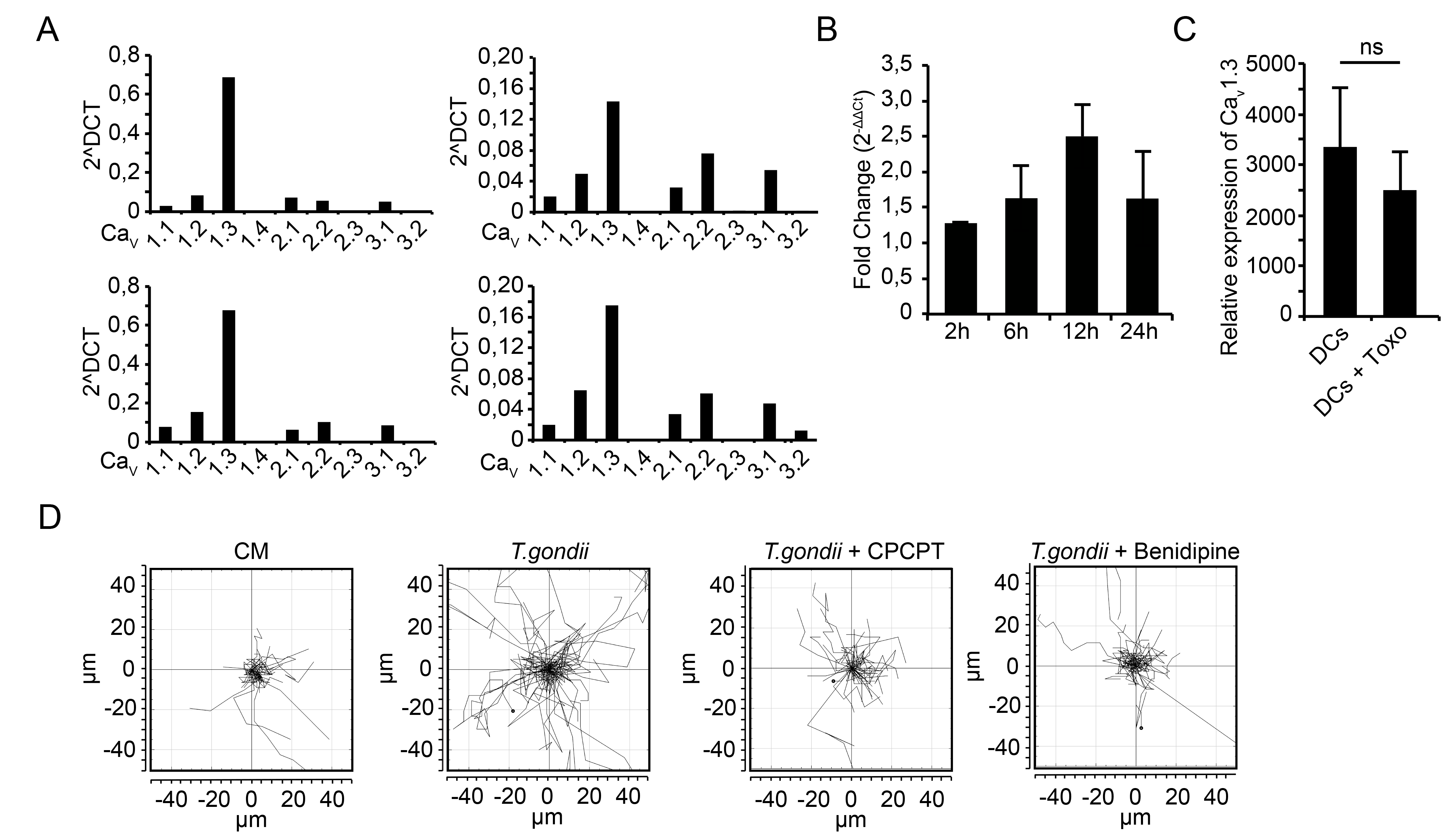

Supplement: S4 Fig — (A) qPCR using primers against α1 subunits of CaV1.1, 1.2, 1.3, 1.4, 2.1, 2.2, 2.3, 3.1 and 3.2 as detailed in Materials and Methods. Each graph depicts expression levels in DCs derived from one individual mouse (n = 4). ΔCt values were calculated with TBP as reference gene. (B) CaV1.3 expression in T. gondii-infected DCs. qPCR analysis of cDNA from DCs challenged with T. gondii tachyzoites (PRU, MOI 3) related to DCs in complete medium at indicated time-points and using primers against the α1 subunit of CaV1.3 as detailed in Materials and Methods. ΔCt values were calculated with TBP as reference gene and are given as means ± SEM of 3 independent experiments performed in triplicate. (C) Ratiometric analysis of Cav1.3 polypeptide expression by western blotting. Bar graph shows, for each condition, the relative expression (mean ± SD) after normalization to internal loading control (GAPDH) from 3 independent experiments (ns: p ≥ 0.05, Student´s t-test). (D) Representative motility plot analysis of DCs in complete medium (CM) or challenged with T. gondii tachyzoites (PTG, 3 h, MOI 3) followed by treatment (1 h) with the selective CaV1.3 inhibitor CPCPT or benidipine, respectively. Data are representative of 3 independent experiments. (TIF) [file ppat.1006739.s007.tif]

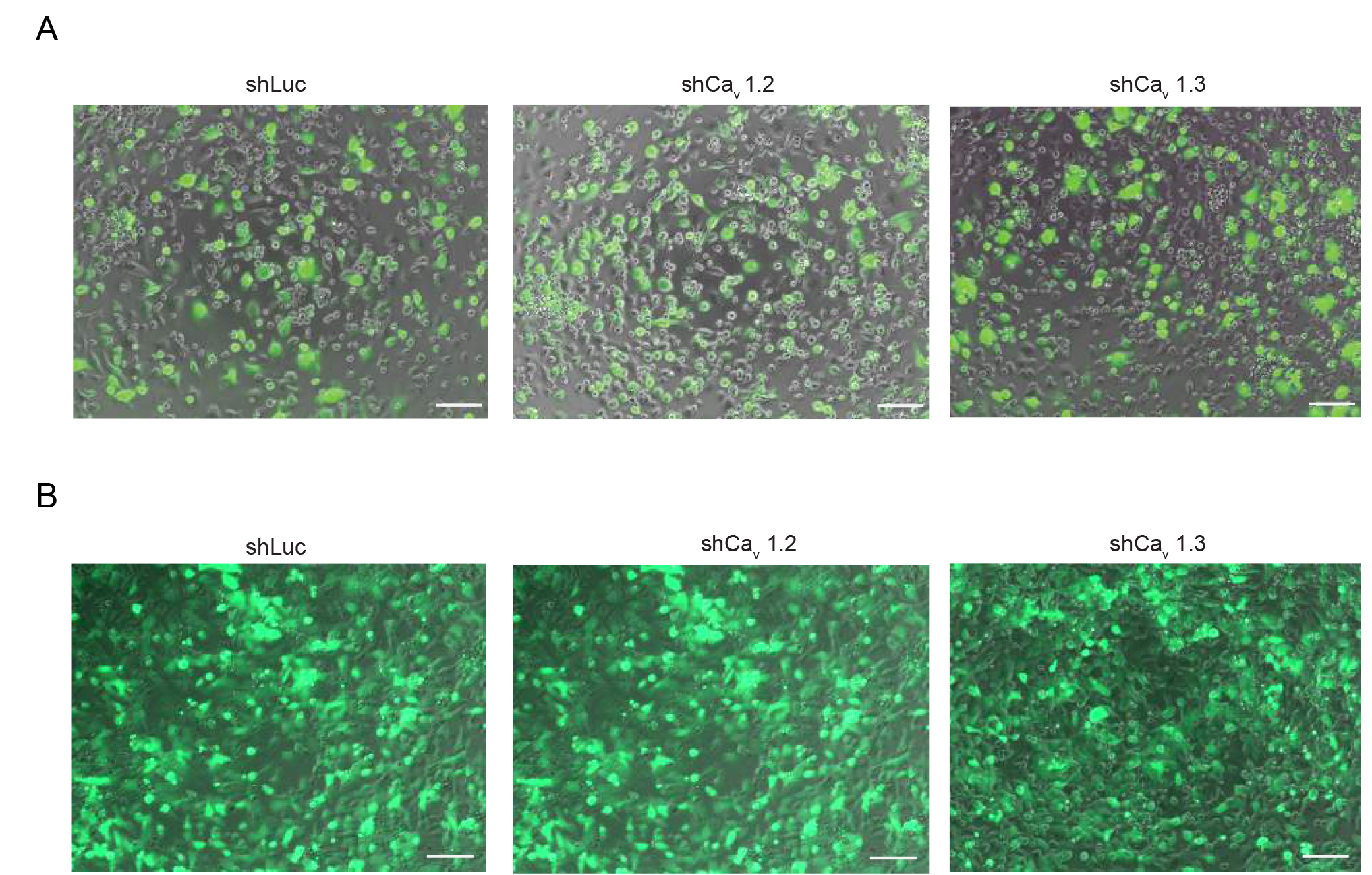

Supplement: S5 Fig — (A and B) Live cell imaging of DCs transduced with EGFP-expressing lentiviral vectors (green) carrying shRNA targeting CaV1.3 (shCav1.3), CaV1.2 (shCav1.2) or a non-related target (Control shRNA, shLuc). Tranductions were performed and evaluated by epifluorescence and light microscopy as indicated under Materials and Methods. The transduction frequencies of cells used in assays were consistently > 50% for primary DCs (A) and > 80% for NE-4C (B). Data are representative of multiple independent transductions. Scale bar: 100 μm. (TIF) [file ppat.1006739.s008.tif]

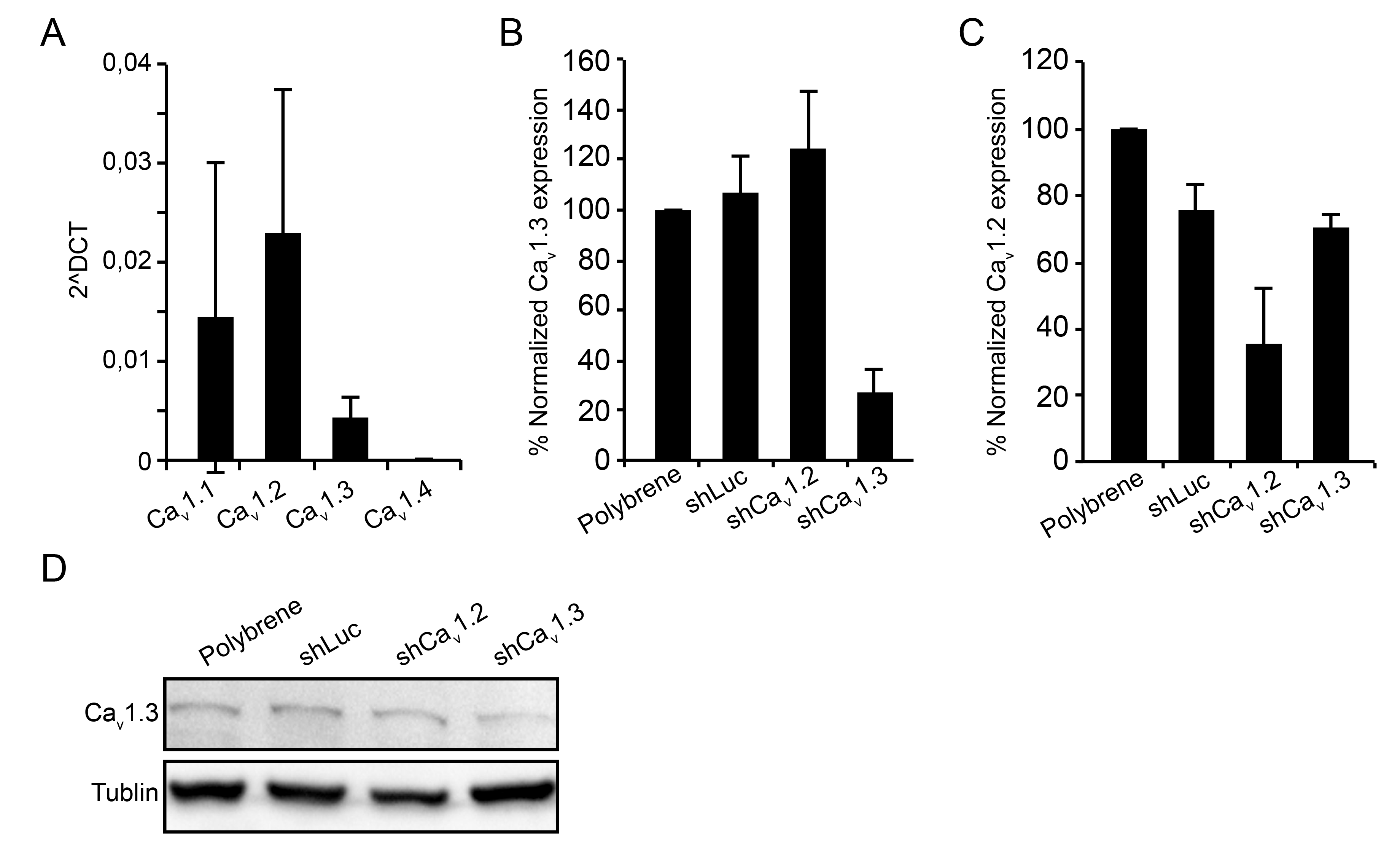

Supplement: S6 Fig — (A) VDCC/Cav1.3expression by the NE-4C line. Compiled qPCR analysis using primers against α1 subunits of CaV1.1, 1.2, 1.3 and 1.4 as detailed in Materials and Methods. ΔCt values are given as means ± SEM of 3 independent experiments performed in duplicate. (B) Relative Cav1.3 expression in NE-4C cells transduced with shCav1.3, shCav1.2 and control shRNA, shLuc related to polybrene-treated DCs assessed by qPCR as indicated under materials and Methods. Data represents means ± SEM of 3 independent experiments. (C) Relative Cav1.2 expression in NE-4C cells transduced with shCav1.2, shCav1.3 and control shRNA, shLuc related to polybrene-treated DCs assessed by qPCR as indicated under materials and Methods. Data represents means ± SEM of 3 independent experiments. (D) Expression of Cav1.3 protein after treatment with polybrene, transduction with shLuc, shCav1.2 and shCav1.3 analyzed by Western blotting as indicated under Materials and Methods. Data is representative of 3 independent experiments. (TIF) [file ppat.1006739.s009.tif]

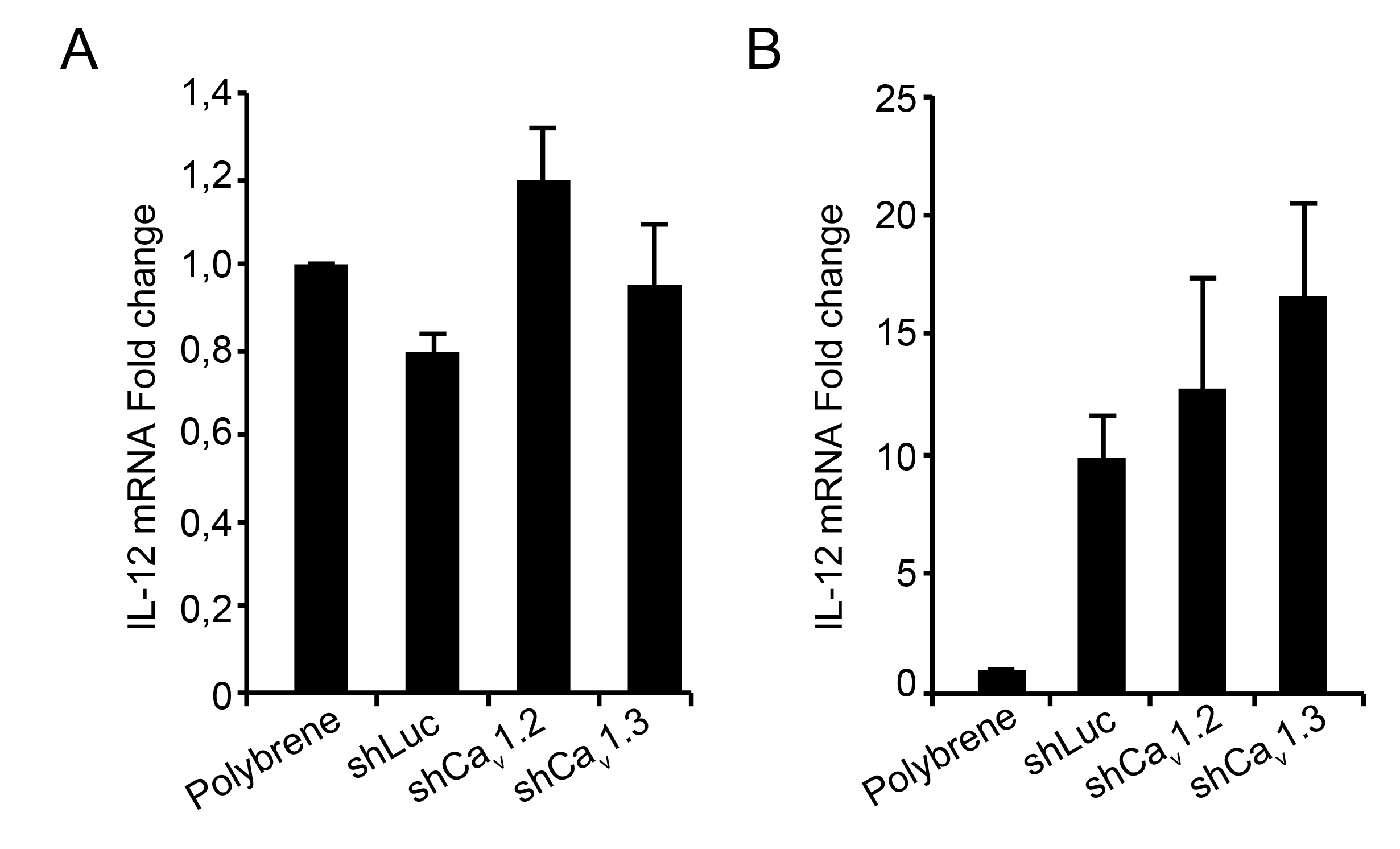

Supplement: S7 Fig — Relative IL-12 mRNA expression in (A) NE-4C cells and (B) primary DCs, respectively, following transduction with shLuc (control), shCav1.2 or shCav1.3 as indicated under Materials and Methods. Data shows fold increased expression related to polybrene treatment (1) and represents means ± SEM of 3 independent experiments performed in duplicate. (TIF) [file ppat.1006739.s010.tif]

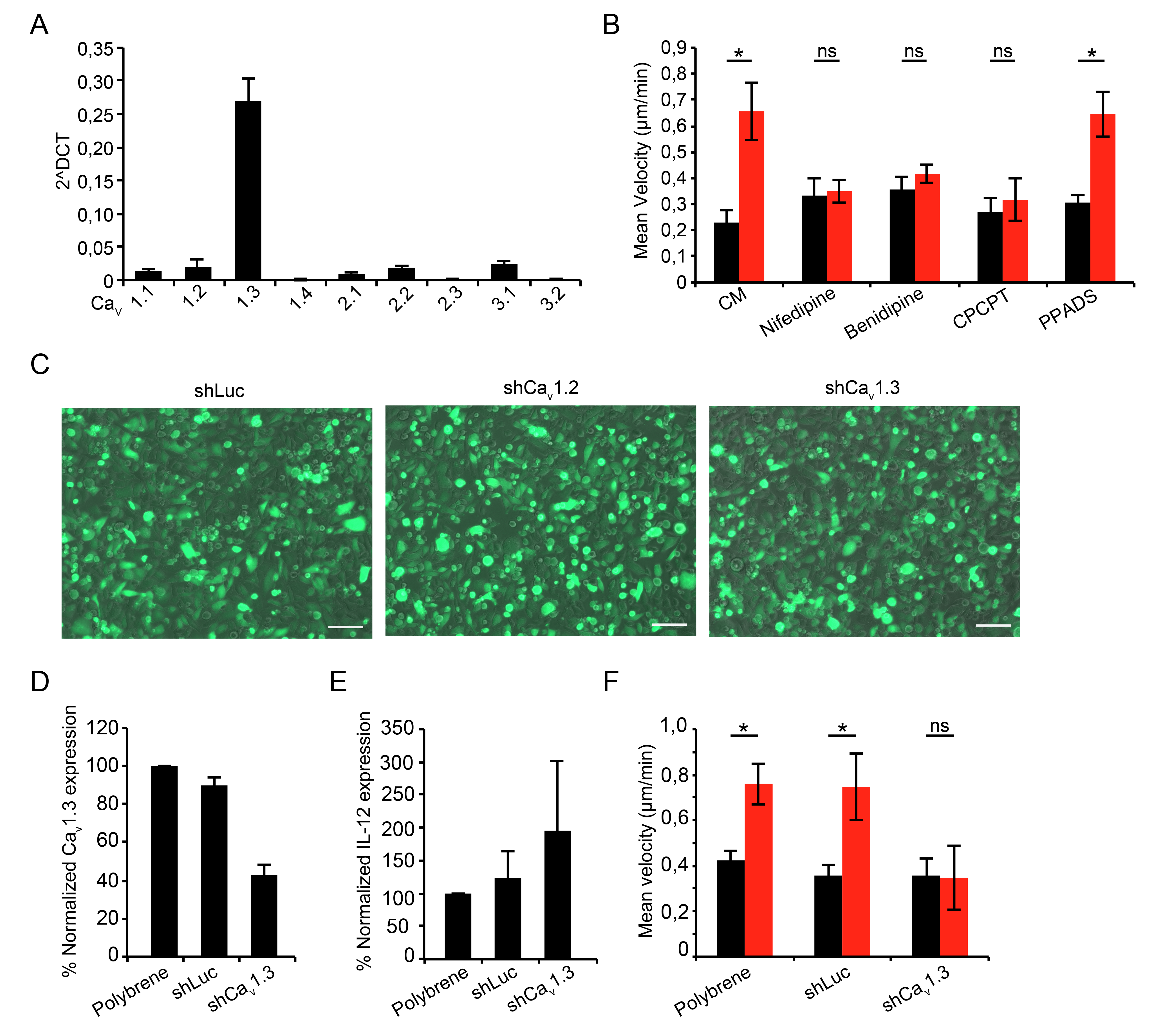

Supplement: S8 Fig — (A) VDCC expression by the JAWS II line. Compiled qPCR analysis using primers against α1 subunits of CaV1.1, 1.2, 1.3, 1.4, 2.1, 2.2, 2.3, 3.1 and 3.2 as detailed in Materials and Methods. ΔCt values are given as means ± SEM of 3 independent experiments performed in duplicate. (B) Motility analysis of JAWS II cells challenged with T. gondii tachyzoites (PTG, 3 h, MOI 3) followed by treatment (1 h) with nifedipine (10 μM), benidipine (10 μM) selective CaV1.3 inhibitor CPCPT (1 μM) or purinergic calcium receptor inhibitor PPADS (100 μM). Data represent median velocities ± SD of 3 independent experiments. Asterisks indicate significant differences (*: p < 0.001, ns: p ≥ 0.05, Pairwise Wilcoxon rank-sum test, Holm correction). (C) Live cell imaging of JAWS II transduced with EGFP-expressing lentiviral vectors (green) carrying shRNA targeting CaV1.3 (shCav1.3) or a non-related target (Control shRNA, shLuc). Tranductions were performed and evaluated by epifluorescence and light microscopy as indicated under Materials and Methods. The transduction frequencies of cells were > 80% for JAWS II. Data are representative of multiple independent transductions. Scale bar: 100 μm. (D) Bar graph shows the relative Cav1.3 expression in JAWS II cells transduced with shCav1.3 and control shRNA (shLuc) related to polybrene-treated DCs assessed by qPCR as indicated under materials and Methods. ata represents means ± SEM of 3 independent experiments. (E) Bar graph (right) shows the relative IL-12 mRNA expression in JAWS II following transduction with shLuc (control) or shCav1.3 as indicated under Materials and Methods. Data shows fold increased expression related to polybrene treatment (1). Data represents means ± SEM of 3 independent experiments. (F) Motility analysis of JAWS II transduced with recombinant lentiviral vectors carrying shRNA targeting Cav1.3 or a non-related target (shLuc). DCs were challenged with T. gondii tachyzoites (PRU, MOI 3). Data represent median velocities ± SD o [file ppat.1006739.s011.tif]

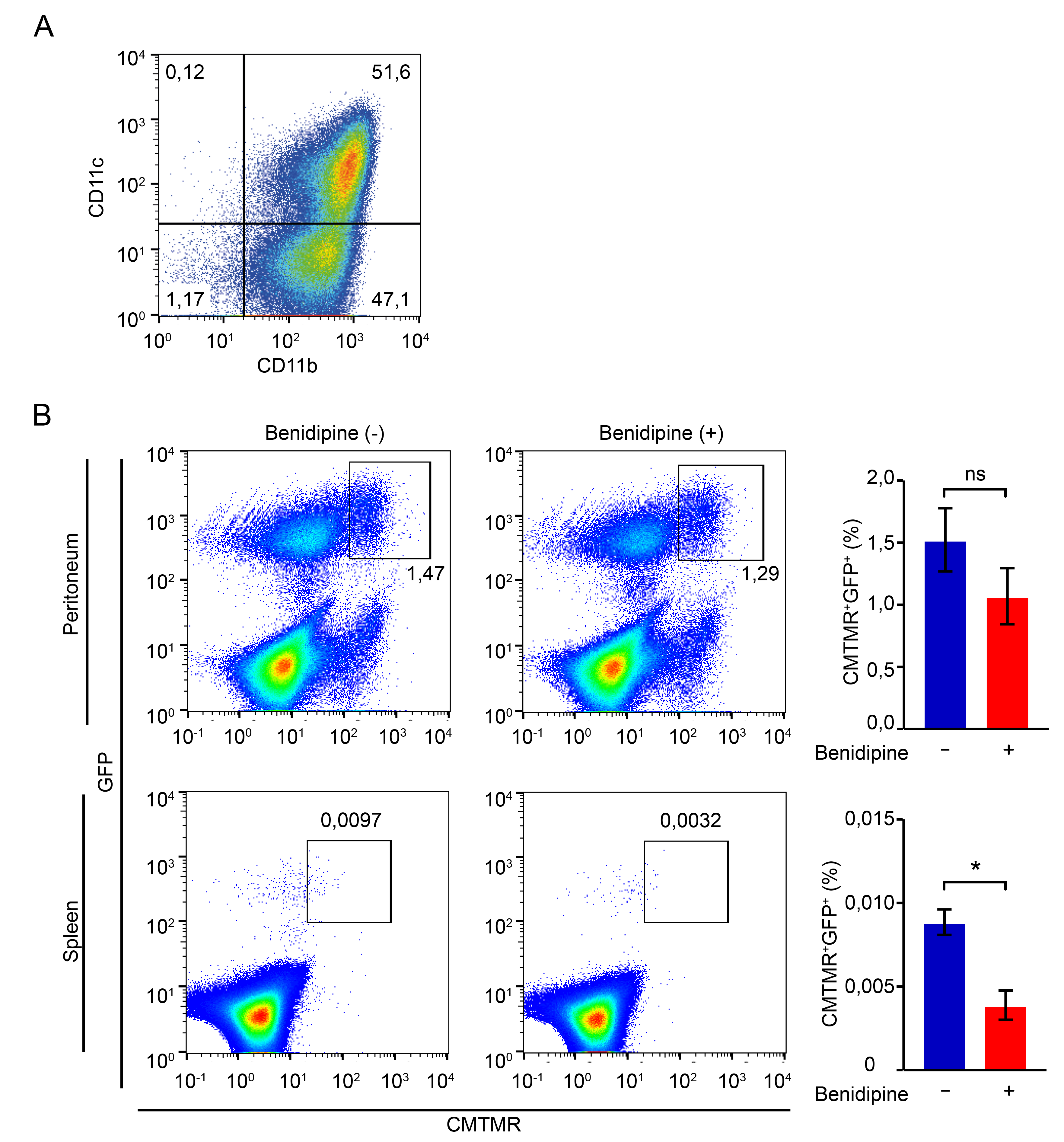

Supplement: S9 Fig — (A) Flow cytometry analysis show the expression of CD11c and CD11b by murine bone-marrow-derived DCs utilized in adoptive transfers, as indicated under Materials and Methods. Plot is representative of multiple independent analyses. (B) Flow cytometry analysis of spleen and peritoneal fluid 24 h post-inoculation of 5x10^6^ T. gondii (GFP-expressing PTGluc)-infected DCs ± benidipine, as indicated under Materials and Methods. Cells were stained with CMTMR previous to inoculation. Representative bivariate dot plots show, for each condition, adoptively transferred parasite-associated DCs (CMTMR+ GFP+) and CMTMR- parasite-associated cells (CMTMR- GFP+). Analysis was gated on live CD3- CD19- GR1-NK1.1- cells. Gatings indicate percentage of CMTMR+ GFP+ cells related to the total cell population. Plots are representative from 3 independent experiments. Bar graphs indicate, for each condition, the percentage of CMTMR+ GFP+ cells from 3 independent experiments (*: p < 0.05; ns: p ≥ 0.05, Student´s t-test, n = 3). (TIF) [file ppat.1006739.s012.tif]

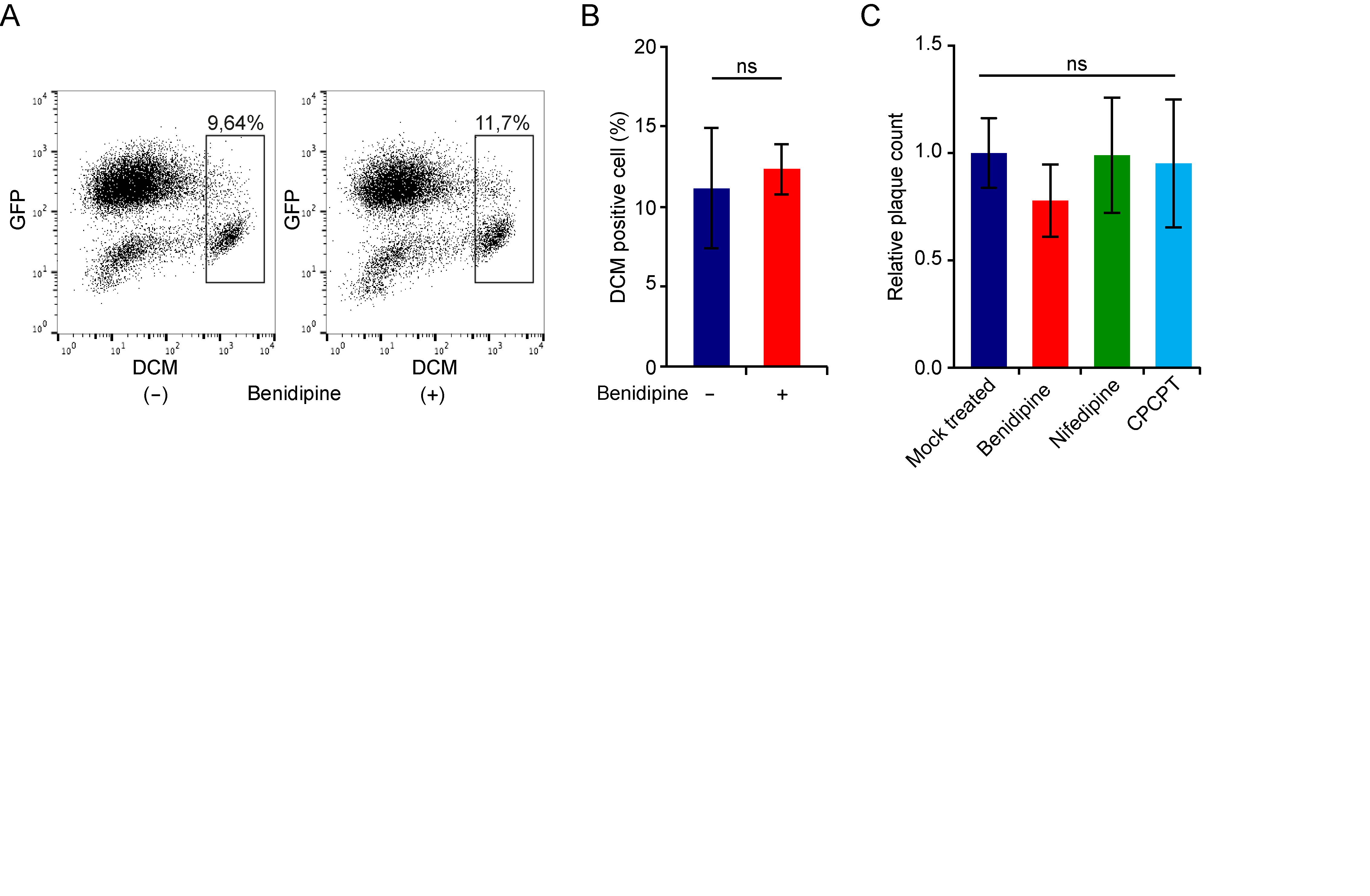

Supplement: S10 Fig — (A) Flow cytometry analysis of DCs challenged with GFP-expressing PTGluc tachyzoites in absence or presence of benidipine as indicated under Materials and Methods. Representative plots show infected DCs (GFP+) and viability (DCM+). (B) Bar graph shows percentage of DCM+ cells in presence or absence of benidipine as in (A). Data represent mean ± SD of 3 independent experiments (ns: p ≥ 0.05, Student´s t-test). (C) Plaquing assays of T. gondii-challenged DCs ± benidipine (40μM), nifedipine (30μM) or CPCPT (10μM). Following challenge with freshly egressed tachyzoites (PTGluc, MOI 3, 6 h) and treatment (3 h post-challenge), DCs were force-lyzed before plaquing as indicated under Materials and Methods. Equal volumes of force-lyzed challenged DC suspensions were plated and plaques were counted by epifluorescence microscopy. Bar graph shows compiled analyses of plaque counts related to the force-lyzed and mock-treated controls with complete medium (plaque counts in the mock-treated control was set to 1). Data represent mean ± SD of 3 independent experiments (ns: p ≥ 0.05, Student´s t-test). (TIF) [file ppat.1006739.s013.tif]
